# *Pistacia lentiscus* L. Distilled Leaves as a Potential Cosmeceutical Ingredient: Phytochemical Characterization, Transdermal Diffusion, and Anti-Elastase and Anti-Tyrosinase Activities

**DOI:** 10.3390/molecules27030855

**Published:** 2022-01-27

**Authors:** Wiem Elloumi, Amina Maalej, Sergio Ortiz, Sylvie Michel, Mohamed Chamkha, Sabrina Boutefnouchet, Sami Sayadi

**Affiliations:** 1 Laboratory of Environmental Bioprocesses, Center of Biotechnology of Sfax, University of Sfax, Sfax 3018, Tunisia; wiem.elloumi@enis.tn (W.E.); maalejamina@yahoo.fr (A.M.); mohamed.chamkha@cbs.rnrt.tn (M.C.); 2 CiTCoM, CNRS 8038, PNAS Team (Natural Products, Analysis and Synthesis), Faculté de Santé, Université de Paris, 4, av. de l’Observatoire, 75006 Paris, France; sergio.ortiz.aguirre@gmail.com (S.O.); sylvie.michel@parisdescartes.fr (S.M.); sabribou2@yahoo.fr (S.B.); 3 Biotechnology Program, Center for Sustainable Development, College of Arts and Sciences, Qatar University, Doha 2713, Qatar

**Keywords:** *Pistacia lentiscus* L. leaves, LC-MS, nuclear magnetic resonance, quercetin-3-*O*-rhamnoside, myricetin-3-*O*-rhamnoside, transdermal diffusion, tyrosinase inhibition, elastase inhibition, cytotoxicity

## Abstract

The present work was performed to investigate the phenolic composition of *P. lentiscus* L. distilled leaves (PDL) and examine its potential against certain key enzymes related to skin aging. High-pressure liquid chromatography coupled to mass spectrometry (HPLC-MS) and various separation procedures combined with nuclear magnetic resonance (NMR) and MS analysis were performed to isolate and identify compounds present in the ethyl acetate extract (EAE) of PDL. A high amount of flavonol glycoside was detected in EAE. Indeed, quercetin-3-*O*-rhamnoside (FC), myricetin-3-*O*-rhamnoside (FM2), and kaempferol-3-*O*-rhamnoside (FB2) were isolated from EAE, and are present in high quantities of 10.47 ± 0.26, 12.17 ± 0.74, and 4.53 ± 0.59 mg/g dry weight, respectively. A transdermal diffusion study was carried out to determine the EAE-molecules that may transmit the cutaneous barrier and showed that FM2 transmits the membrane barrier with a high amount followed by FC. EAE, FM2, and FC were tested against tyrosinase and elastase enzymes. Moreover, intracellular tyrosinase inhibition and cytotoxicity on skin melanoma cells (B16) were evaluated. The results indicated that EAE, FC, and FM2 have important inhibitory activities compared to the well-known standards, at non-cytotoxic concentrations. Therefore, they could be excellent agents for treating skin pigmentation and elasticity problems.

## 1. Introduction

Skin is exposed to several intrinsic and extrinsic factors that adversely affect its natural and youthful appearance. Intrinsic factors are associated with the natural aging of the skin, which is stimulated by changes in skin elasticity and hereditary genes. However, extrinsic factors are mainly caused by ultraviolet (UV) rays, drugs, burns, and injuries. These triggers may result in unequal pigmentation, brown spots, wrinkles, and loss of skin elasticity.

Skin hyperpigmentation is caused by the over biosynthesis of melanin that occurs in melanocytes within the basal epidermis [1]. Indeed, tyrosinase, the key enzyme in melanin formation, catalyzes the hydroxylation of l-tyrosine to 3,4-dihydroxyphenylalanine (l-DOPA) and subsequent oxidation of l-DOPA to dopaquinone [2,3]. The high concentration of this pigment in the cutaneous tissues generates reactive oxygen species (ROS) and hydrogen peroxide (H_2_O_2_) [4]. Melanogenesis is mainly induced by high UV exposure, drugs, burns, or post-inflammatory conditions [5].

On the other hand, elastin, a major fiber of the extracellular matrix (ECM), provides recoil to tissues that undergo repeated stretch [6]. Commonly, the synthesis of elastin fibers decreases with age and is replaced by inextensible collagen [7]. Moreover, overexposure to UV radiation decreases the synthesis of elastin fiber and increases the production of ROS [8]. As a result, loss of skin elasticity, wrinkles, and stretch marks appear as skin aging problems [9]. Indeed, the elastase enzyme is responsible for the breakdown of elastin as well as collagen, fibronectin, and other ECM proteins [10].

Tyrosinase and elastase inhibitors are widely used in medicinal and cosmetic products due to their antioxidant effects and their ability to reduce hyperpigmentation [11] and skin elasticity problems, respectively. However, commercial skin-care products can induce adverse reactions such as skin irritation, unequal pigmentation, and even cancer [4]. Thus, it remains necessary to develop natural, safe, and more effective skin-care products.

Nowadays, natural compounds extracted from medicinal and aromatic plants are of great pharmaceutical interest due to their potent biological properties. One of the main potentials of medicinal and aromatic plants is the production of essential oils. However, the ratio between the oil production and the plant biomass processed is very low, which generates important amounts of by-products whose management must be taken into account [12]. 

*Pistacia lentiscus* L. is an evergreen tree widely distributed in the Mediterranean region [13,14]. It belongs to the Anacardiaceae family, which includes about 70 genera and over 600 species. In Tunisia, *P. lentiscus* L. has a wide geographical repartition extending from the North to the South [15]. The fruits, leaves, and resins of *P. lentiscus* L. have a long tradition in ancient medicine. They have been used for the treatment of eczema and jaundice as skin diseases, as well as, gastrointestinal complaints, throat infection, and renal stone [16]. The aerial part of *P. lentiscus* and its essential oil fraction has been applied for the repair of wounds and burns and treatment of skin diseases [17]. The most ancient uses of *P. lentiscus* leaves date back to the Nuragic civilization and have been attributed to the Sardinian population, which used the water extract as a wound-healing agent [18].

*P. lentiscus* L. was found to possess anti-inflammatory, anticancer [19,20], antioxidant [21], anti-aging, skin-care [22,23], anti-proliferative [24], and antimicrobial [25] properties. These pharmacological effects of *P. lentiscus* L. are due to the presence of secondary metabolites belonging to several chemical classes such as anthocyanins, flavonoids, phenolic acids, triterpenoids, and tannins [26,27]. Indeed, the quality of these secondary metabolites depends mainly on the technological processes involved in their extraction and isolation [28,29].

In previous studies, there is no detailed research on the therapeutic potential of the bioactive molecules extracted from the by-product of the distillation of the leaves of *P. lentiscus*. If only in Tunisia the annual production of essential oil from the leaves of *P. lentiscus* was 100 L, this implies the co-generation of more than 100 tons/year of solid waste which can be valorized [30]. In this context, the present study was performed to determine the phenolic profile of the active extract of *P. lentiscus* L. distilled leaves (PDL) and purify its main molecules to evaluate their potential as skin whitening and skin elasticity agents. Indeed, the phenolic compounds, that transmit the membrane barrier, were evaluated against tyrosinase and elastase enzymes, and also for their intracellular tyrosinase and cytotoxicity activities on skin melanoma cells (B16).

## 2. Results

### 2.1. Total Phenolic Content (TPC)

The methanol extract (ME) of PDL obtained by pressurized solvent extraction (PSE) and its ethyl acetate (EAE) and aqueous (AE) phases were evaluated for their TPC using Folin–Ciocalteu reagent. Indeed, the ME was separated into two phases (EAE and AE) to distinguish the most active extract and subsequently simplify the step of molecules isolation. The amounts of phenolic compounds present in PDL extracts were recapitulated in Table 1. Results indicate that EAE is significantly richer in phenolic compounds than AE (*p* < 0.001).

### 2.2. Total Flavonoid Content (TFC)

The TFC of ME, EAE, and AE was determined using the method of aluminum chloride (AlCl_3_). Results showed that EAE is significantly richer in flavonoid compounds compared to the AE fraction (*p* < 0.0001) (Table 1).

### 2.3. Antioxidant Capacity

#### 2.3.1. DPPH Radical Scavenging Assay

The 1,1-phenyl-2-picrylhydrazyl (DPPH) radical scavenging potential of PDL extracts showed a good antioxidant activity compared to the standard Ascorbic acid (IC_50_ = 13.85 µg/mL). The extracts have broadly similar IC_50_ values with an average of 19 µg/mL (Table 2). The lowest IC_50_ value of 18.07 µg/mL was obtained by EAE.

#### 2.3.2. Ferric Reducing Antioxidant Power Assay (FRAP)

The FRAP assay was used to evaluate the reducing power of PDL extracts. Values were expressed as Ascorbic acid equivalents (AEAC) and summarized in Table 2. It was found that EAE (522.76 ± 22.99 mg AEAC/g of extract) is significantly more active than AE and ME (*p* < 0.0001).

The results of TPC, TFC, and antioxidant activities confirmed that EAE has a rich phenolic antioxidant composition compared to AE. Indeed, EAE corresponds to ME but with a minimum of polar compounds, which simplifies the step of compounds isolation. Thus, EAE has been selected to be chemically analyzed to determine its phenolic composition.

### 2.4. High-Pressure Liquid Chromatography Coupled to Mass Spectrometry (HPLC-MS) Analysis

**Figure 1:**
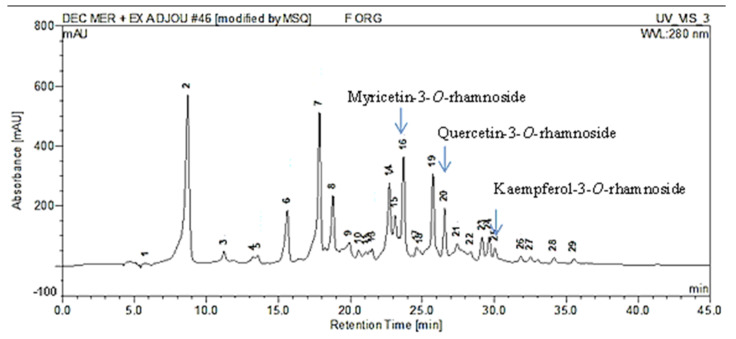
Chromatographic profile of the ethyl acetate extract (EAE) of *P. lentiscus* L. distilled leaves (PDL) acquired by HPLC-MS, detected at 280 nm. The three major isolated molecules from this extract were mentioned in the figure.

HPLC-MS analysis was performed as the first step for phenolic compounds identification by comparing our molecular ions peaks data and UV/Vis absorption maxima (λ_max_) data with other publications. The HPLC-MS chromatogram of EAE, recorded at 280 nm, shows a rich composition in phenolic compounds eluted from 8 to 35 min (Figure 1).

**Figure 2:**
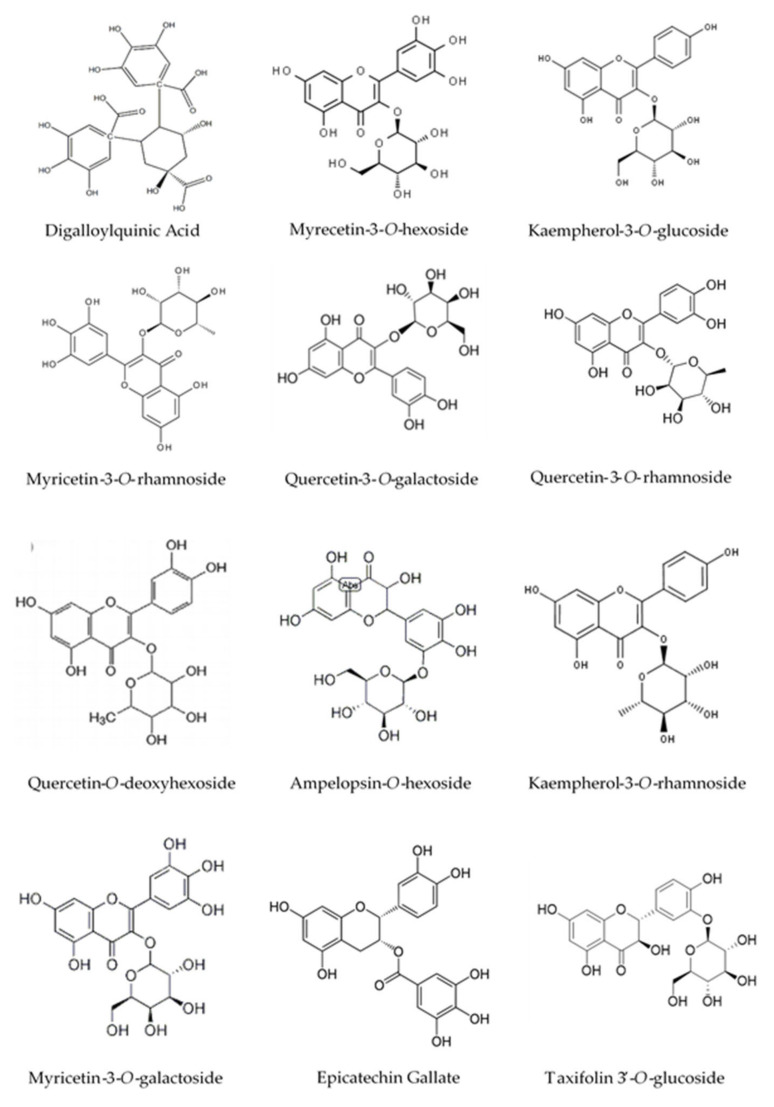
Structure of the constituents identified in the ethyl acetate extract (EAE) by HPLC-MS.

As figured in Table 3, our extract has a high level of flavonol glycosides. The ionization in the negative mode of myricetin-3-*O*-galactoside, myricetin-3-*O*-hexoside, myricetin-3-*O*-rhamnoside, and myricetin-*O*-galloyl-deoxyhexoside showed a signal at *m*/*z* 479, 479, 463, and 615, respectively. The similarity of the negative ion mass spectrum [M − H]^−^ and the UV spectra of these molecules (Table 3) with those found by de Brito et al. [31] and Saldanha et al. [32] confirms their structures. Peaks 17, 20, and 21 were identified as quercetin-3-*O*-galactoside, quercetin-3-*O*-rhamnoside, and quercetin-*O*-deoxyhexoside, respectively by comparing our results with those reported by Pacifico et al. [33]. Peak 15 and 26 with [M − H]^−^ (*m*/*z*) 447 and 431 were identified as kaempferol-3-*O*-glucoside and kaempferol-3-*O*-rhamnoside, respectively. The structure of the molecules identified by HPLC-MS were presented in Figure 2. 

### 2.5. Purification and Quantification of Phenolic Compounds

Various chromatographic procedures were applied to isolate and purify the EAE compounds, which were then identified by comparing the nuclear magnetic resonance (NMR) spectroscopy data (1D-NMR, 2D-NMR) (400 MHz, in deuterated methanol (MeOD-*d*_4_)) and the mass spectrometry (MS) data with those of previous publications.

EAE was firstly fractionated by centrifugal partition chromatography (CPC) technique. The solvents system used for the CPC separation was selected after the determination of the *Rf* value. A 60 F254 gel-coated glass sheet (TLC) of EAE side by side with a standard flavonol glycoside available in the laboratory (quercetin-3-*O*-rhamnoside) was developed to determine its *Rf*. Then, the spot putatively associated with this compound was selected for the CPC experiment. This spot was chosen for estimation of the partition coefficient (K) and selection of solvents system by the shake-flask method. Four fractions, namely A, B, C, and M, have been obtained after the CPC fractionation of EAE.

**Figure 3:**
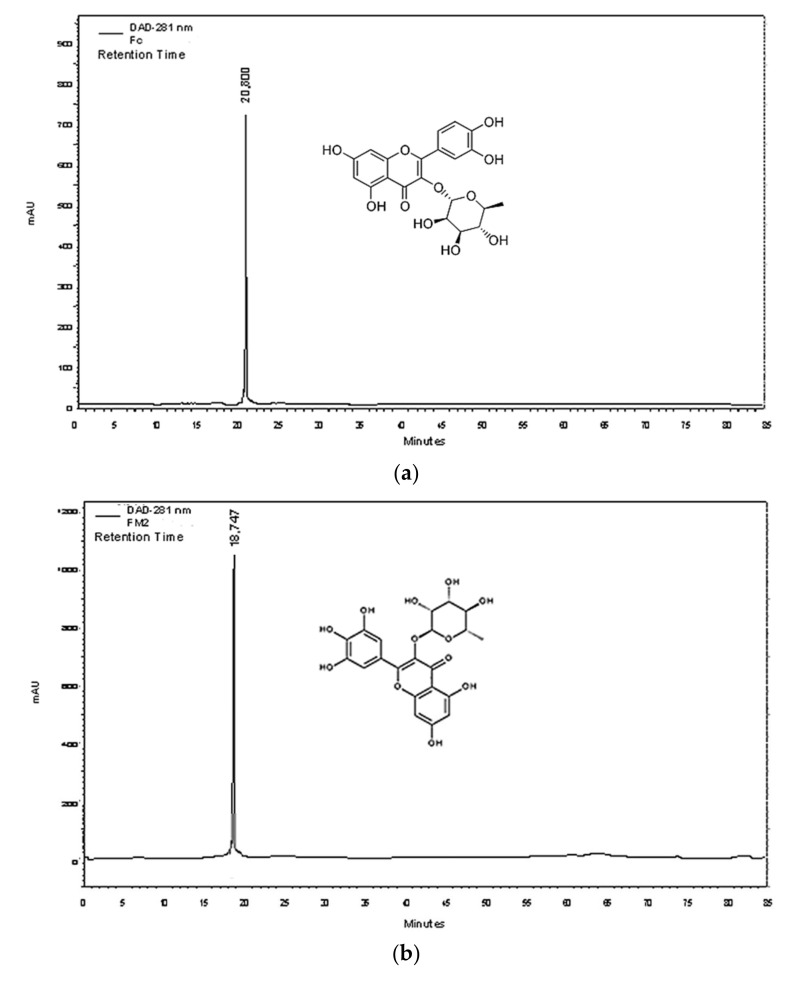

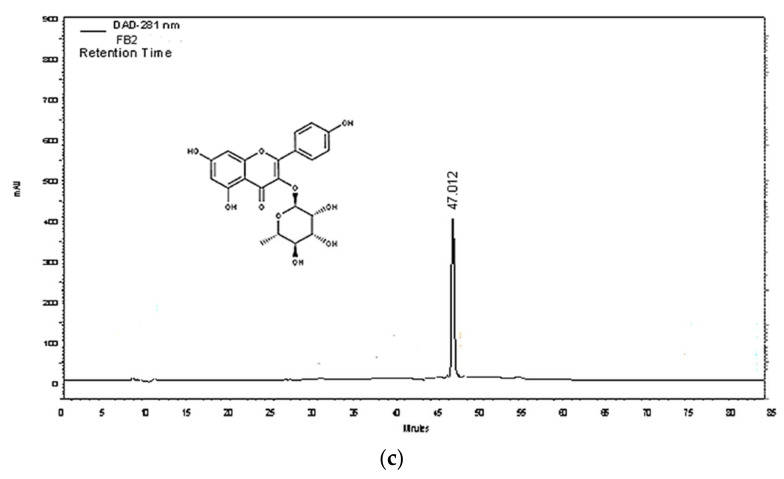
Chromatographic profile of (**a**) C fraction (Quercetin-3-*O*-rhamnoside), (**b**) M2 fraction (myricetin-3-*O*-rhamnoside), and (**c**) B2 fraction (kaempferol-3-*O*-rhamnoside) acquired by HPLC-DAD.

The C fraction (0.22 g) is a pure molecule isolated in a single CPC step. Its purity was verified by HPLC-DAD analysis (Figure 3a). 

**Figure 4:**
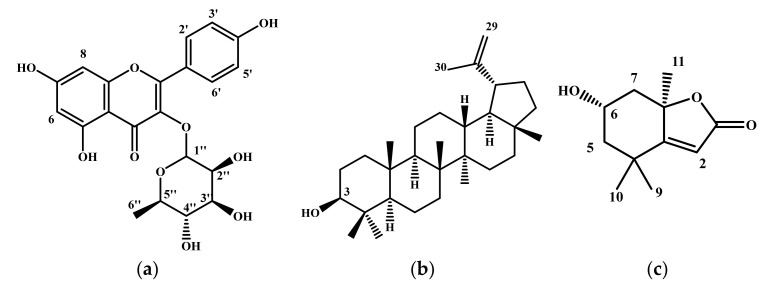
The general structures of molecules isolated and identified in EAE; (**a**) Structure of flavonol glycosides, (**b**) Structure of lupeol, and (**c**) Structure of loliolide.

The NMR spectroscopy data in 1D and 2D of the C fraction (Figure 4a and in Supplementary Material Figures S1–S4 and Table S1) are similar to those found by Aderogba et al. [34]. The mass spectrum of the ionization in the negative mode of this molecule showed a signal at *m*/*z* 447 (C_21_H_20_O_11_) (Supplementary Material Figure S5). Thus, it was identified as quercetin-3-*O*-rhamnoside or quercitrin.

The HPLC profile of the A fraction (3.41 g) demonstrated that this fraction can be more purified. It was fractionated by preparative HPLC using the E1 solvent gradient. According to the NMR spectra of the obtained fractions, only the A12 fraction (8 mg) and the A32 fraction (9 mg) were pure and were structurally identified. The NMR data of the A12 fraction (Figure 4b and in Supplementary Material Figures S6–S9 and Table S2) are similar to those found by Katekhaye et al. [35]. The molecular ion of the ionization in the negative mode of this molecule is (*m*/*z*) 425 (Supplementary Material Figure S10). From those findings, the A12 molecule was identified as lupeol. Besides, the NMR spectroscopy data of the A32 molecule (Figure 4c and in Supplementary Material Figures S11 and S12 and Table S2) are in agreement with the literature [36]. The mass spectrum data of the ionization in the negative mode of A32 showed a signal at (*m*/*z*) 195 (Supplementary Material Figure S13). Thus, the A32 molecule was identified as loliolide.

HPLC analysis of the M fraction (0.18 g) showed that this fraction was composed of more than two molecules. The M2 fraction (0.034 g) was obtained after fractionation of the M fraction by the Medium Pressure Liquid Chromatography (MPLC) technique. The purity of this fraction was confirmed by HPLC analysis (Figure 3b). Our NMR spectroscopy data in 1D and 2D (Figure 4a and in Supplementary Material Figures S14 and S15 and Table S1) are in agreement with those found by Braca et al. [37]. The mass spectrum of the ionization in the negative mode of M2 showed a signal at *m*/*z* 463 (C_21_H_20_O_12_) (Supplementary Material Figure S16). M2 molecule was identified as myricetin-3-*O*-rhamnoside.

Finally, the HPLC profile of the B fraction (0.22 g) indicated that this fraction can be more purified. Two fractions were obtained, B1 (12 mg) and B2 (7 mg), after fractionation by preparative HPLC using E2 solvent gradient. HPLC-DAD profiles of B1 and B2 demonstrated that both of them are pure. The 1H-NMR spectra and MS data of the B1 molecule are similar to those of the C molecule. It was identified as quercitrin. The NMR data in 1D and 2D of B2 molecule (Figure 4a and in Supplementary Material Figures S17–S20 and Table S1) are similar to those found by Xu et al. [38]. This molecule has a molecular ion of the ionization in negative mode (*m*/*z*) at 431 (Supplementary Material Figure S21). This molecule was identified as kaempferol-3-*O*-rhamnoside (Figure 3c).

The HPLC-DAD method has been validated for the quantification of isolated molecules. It is an external validation, where the standards used are the pure molecules isolated from the extract which are quercetin-3-*O*-rhamnoside (FC), myricetin-3-*O*-rhamnoside (FM2), and kaempferol-3-*O*-rhamnoside (FB2). Three concentration levels (low, medium, and high) were evaluated for each molecule. The analysis generated a standard curve for each isolated compound, whose regression equations and linear correlation coefficients (r^2^) are summarized in Table 4 (Y and X are the area of the peak and the concentration respectively). Indeed, the r^2^ coefficient for each compound is greater than 0.99 and the relative standard deviation (RSD) percentages at different levels (low, medium, and high) are less than 5%, as described in RDC 166/2017. The accuracy percentages of each molecule at different levels are greater than 96% (Table 4). Therefore, the HPLC method is validated for FC, FM2, FB2 quantification. Quantification of FC, FM2, and FB2 in PDL showed that they contain a high amount of FM2 (12.173 ± 0.742 mg/g dry weight), followed by FC then FB2 (Table 4).

### 2.6. Transdermal Diffusion

**Figure 5:**
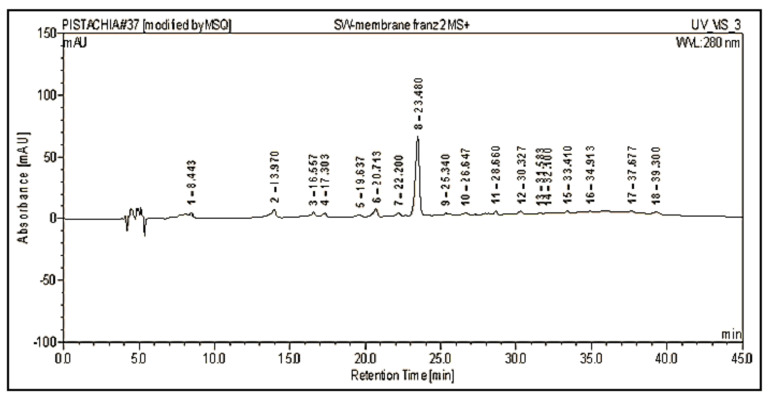
Chromatographic profile of the receptor medium of Franz cell after diffusion of 5% EAE-hydro-cream formulation through the start-M membrane, acquired by HPLC-MS at 280 nm. The peak assignments are listed in Table 3.

The transdermal diffusion of 5% EAE-hydro-cream formulation was evaluated using Franz diffusion cell and start-M membrane. This technic is comparable to the cutaneous absorption of the penetrants. The receptor medium of the Franz cell was analyzed by HPLC-MS to determine the bioactive compounds that crossed the strat-M membrane after 24 h of incubation. Results showed that digalloyl quinic acid ([M − H]^−^ *m/z* 495, λ_max_ 219, 276 nm), myricetin-3-*O*-rhamnoside ([M − H]^−^ *m*/*z* 463, λ_max_ 263, 350 nm), quercetin-3-*O*-galactoside ([M − H]^−^ *m*/*z* 463, λ_max_ 269, 351 nm), and quercetin-3-*O*-rhamnoside ([M − H]^−^ *m*/*z* 447, λ_max_ 258, 351 nm) have crossed the membrane in varying amounts (Figure 5). The bioactive molecules FC and FM2 that crossed the strat-M membrane were subjected to quantification. Results showed that FM2 is the highest molecule crossing the start-M membrane with a quantity of 52.102 ± 0.001 µg/g of 5% hydro-cream. Moreover, FC has crossed the membrane but with a lower concentration than FM2 (Table 4).

### 2.7. Enzymatic Inhibition Activities In Vitro

#### 2.7.1. Tyrosinase Inhibition Assay

**Figure 6:**
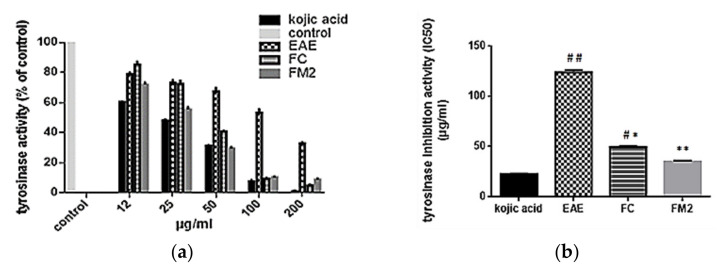
(**a**) Tyrosinase activity (% of control) and (**b**) tyrosinase mushroom inhibition activity (IC_50_) of ethyl acetate extract (EAE), quercetin-3-*O*-rhamnoside (FC), myricetin-3-*O*-rhamnoside (FM2), and kojic acid (standard). (Bars, ± SD (*n* = 3)), * significant *p* ˂ 0.001 and ** significant *p* ˂ 0.0001 compared to EAE, # significant *p* ˂ 0.001 and ## significant *p* ˂ 0.0001 compared to kojic acid.

EAE and its pure molecules FC and FM2 were evaluated in vitro for their tyrosinase inhibition activity. Results indicated that pure molecules have higher tyrosinase inhibition activity compared to EAE, and have near activity compared to the standard kojic acid. As indicated in Figure 6a, mushroom tyrosinase activity decreases significantly in dose depending manner with EAE, FC, and FM2. At a concentration of 100 µg/mL, the percentages of tyrosinase activity with EAE, FC, and FM2 were equal to 53%, 9%, and 10%, respectively. While the percentage of tyrosinase activity with Kojic acid was 7%. The IC_50_ value of tyrosinase activity of EAE (123 µg/mL) was significantly higher compared to that of FM2 (34 µg/mL) and FC (49 µg/mL) (*p* < 0.001). No significant difference was marked between IC_50_ values of FM2 and kojic acid (20 µg/mL) (*p* > 0.05) (Figure 6b).

#### 2.7.2. Elastase Inhibition Assay

**Figure 7:**
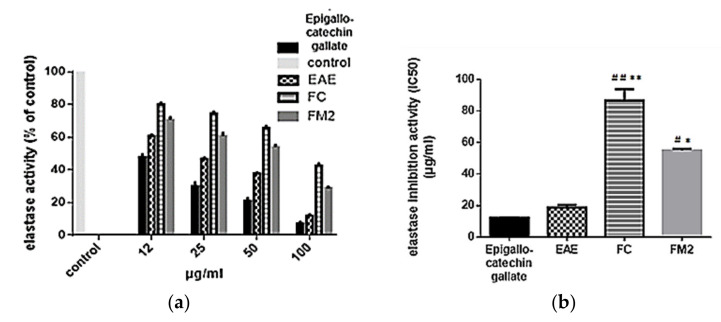
(**a**) Elastase activity (% of control) and (**b**) elastase inhibition activity (IC_50_) of ethyl acetate extract (EAE), quercetin-3-*O*-rhamnoside (FC), myricetin-3-*O*-rhamnoside (FM2), and Epigallocatechin gallate (standard). (Bars, ± SD (*n* = 3)), * significant *p* ˂ 0.001 and ** significant *p* ˂ 0.0001 compared to EAE, # significant *p* ˂ 0.001 and ## significant *p* ˂ 0.0001 compared to Epigallocatechin gallate.

The elastase inhibition activity of EAE and its pure molecules FC and FM2 was evaluated at different concentrations ranging from 12 to 100 µg/mL. Results indicated that EAE has higher elastase inhibition activity compared to the pure molecules, and has a near activity compared to the standard Epigallocatechin gallate. As indicated in Figure 7a, the elastase activity decreases significantly in dose depending manner with EAE, FC, and FM2. At a concentration of 100 µg/mL, the percentages of elastase activity with EAE, FC, and FM2 were equal to 12%, 42%, and 29%, respectively. Besides, the percentage of elastase activity with Epigallocatechin gallate was 8%. The IC_50_ values of elastase activity of FC (86 µg/mL) and FM2 (45 µg/mL) were significantly higher compared to that of EAE (19 µg/mL) (*p* < 0.001). No significant difference was marked between IC_50_ values of EAE and Epigallocatechin gallate (12 µg/mL) (Figure 7b).

### 2.8. Cellular Tyrosinase Inhibition Activity on B16 Cells

#### 2.8.1. Cell Viability

**Figure 8:**
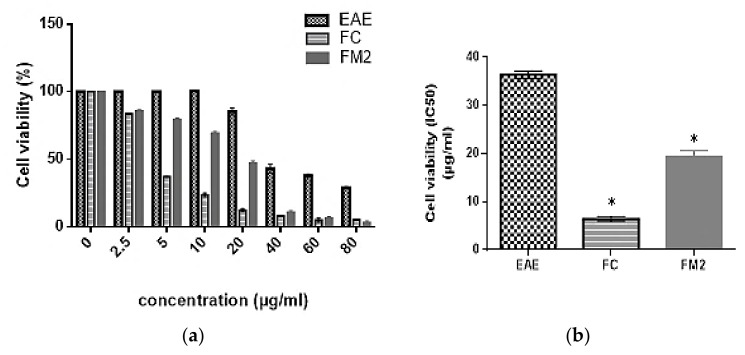
Cytotoxic effect of ethyl acetate extract (EAE), quercetin-3-*O*-rhamnoside (FC), and myricetin-3-*O*-rhamnoside (FM2) on B16 cells (**a**) at different concentrations and (**b**) their IC_50_ values. (Bars, ± SD (*n* = 3)), * significant *p* ˂ 0.0001 compared to EAE.

The cell viability of EAE and the two major isolated molecules FC and FM2 were evaluated against B16 cells. According to ISO 10993-5 [39], a sample with cell viability above 70% is considered non-toxic. Results showed that EAE, FC, and FM2 did not decrease the percentage of cell viability at concentrations below and equal to 20, 2.5, and 10 µg/mL, respectively (Figure 8a). Although, EAE has a cytotoxic effect on B16 cells with an IC_50_ value of 36.39 ± 0.74 µg/mL. The cytotoxic effect of the purified compounds FC and FM2 increases with increasing concentration, with IC_50_ values of 6.40 ± 0.53 and 19.33 ± 1.22 µg/mL, respectively. These values are significantly higher compared to the extract EAE (*p* < 0.0001) (Figure 8b).

#### 2.8.2. Cellular Tyrosinase Inhibition Activity

**Figure 9:**
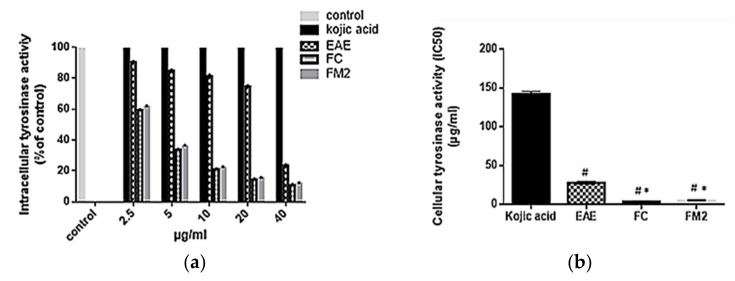
(**a**) Cellular tyrosinase activity (% of control) and (**b**) cellular tyrosinase inhibition activity (IC_50_) on B16 cells of ethyl acetate extract (EAE), quercetin-3-*O*-rhamnoside (FC), myricetin-3-*O*-rhamnoside (FM2), and kojic acid standard (Bars, ± SD (*n* = 3)). * Significant *p* ˂ 0.001 compared to EAE and # significant *p* ˂ 0.0001 compared to kojic acid.

The cellular tyrosinase inhibition activity on B16 cells was evaluated using EAE, FC, and FM2. As indicated in Figure 9a, EAE, FC, and FM2 reduce significantly tyrosinase activity on B16 cells in a dose-dependent manner. Pure molecules FC and FM2 have significantly higher inhibition activity with IC_50_ values of 3.36 ± 0.54, and 4.60 ± 0.47 µg/mL, respectively compared to EAE, which has an IC_50_ value of 27.85 ± 1.75 µg/mL (*p* < 0.001). All of them were even much stronger than the positive control kojic acid (IC_50_ of 142.09 ± 2.72 µg/mL) (*p* < 0.0001) (Figure 9b).

## 3. Discussion

The great interest in the therapeutic effects of medicinal and aromatic plants has been inspired by its traditional use in folk medicine. Currently, the use of bioactive compounds extracted from medicinal plants has stimulated the development of new extracting processes that have an impact on their quality and biological activities [14]. Our work was set up to isolate and identify the phenolic compounds of the Tunisian PDL and assess their contribution to anti-aging and skin whitening properties.

The extraction yield obtained by PSE with methanol was about 23.4% (*w*/*w*), which is four times higher than the one found by Remila et al. [19] using maceration with ethanol. PSE is a green technology based on the use of organic solvents subjected to high temperatures and relatively high pressure [40]. It can therefore be regarded as an effective method for extracting high-yield of phenolic compounds. The ME was separated into two phases (EAE and AE) to simplify the step of molecules isolation. Our results showed that the EAE of PDL has significant TPC and TFC and high antioxidant activities (DPPH and FRAP) compared to ME and AE fractions. Our DPPH results are comparable to those found by Gardeli et al. [41]. Interestingly, our FRAP values are higher than those found by Ben Ahmed et al. [42]. These findings suggest that EAE is rich in phenolic antioxidant compounds derived from PDL. Therefore, this extract was chosen for further chemical analysis.

The HPLC-MS analysis showed that the EAE extract exhibits a high content of flavonol glycosides, including quercetin and myricetin derivatives. This result is consistent with that found in previous research [43]. As reported by Liu et al. [44], the flavonol glycosides group has a good antioxidant activity compared to the standard ascorbic acid, thanks to its chemical structure. This class consists of substances containing one hexose group and two benzene rings (A and B) bound by a pyran chain. The structure-antioxidant activity relationships indicated that the -OH group in 4′ position on the B ring and the -OH group in 7 positions on the A ring possessed high antioxidant activity [45]. Thus, we can say that the flavonol glycosides present in EAE are responsible for its antioxidant activity.

The EAE was then fractionated to isolate its phenolic compounds. Indeed, various separation methods were carried out in this study. The most important one is the CPC technique, which is an eco-friendly purification procedure that leads to compound-rich fractions. As a result, five molecules were isolated from EAE and identified by NMR and MS, namely quercetin-3-*O*-rhamnoside (FC), myricetin-3-*O*-rhamnoside (FM2), kaempferol-3-*O*-rhamnoside (FB2), lupeol, and loliolide. Loliolide molecule, a C11-terpene lactone with the molecular formula C_11_H_16_O_3_, has already been isolated from a large number of plant species mainly families of Phanerogamae [46] and several algae [47]. To the best of our knowledge, this molecule has been identified in the *P. lentiscus* L. plant, for the first time in this study. Lupeol, a triterpenoid derived from lupane with the molecular formula C_30_H_50_O, has been previously identified in *Pistacia lentiscus* var. Chia mastic gum by GC-MS [48]. Furthermore, FC, FM2, and FB2 molecules have previously been identified in *P. lentiscus* L. leaves. Interestingly, our study revealed that these molecules are found in higher levels in the Tunisian *P. lentiscus* L. leaves compared to those in the leaves of *P. lentiscus* L. from Southern Tuscany Italy [49]. Consequently, these molecules may contribute significantly to the antioxidant activity of EAE.

Humans have always been desired to extend their health and youthful appearance by using natural compounds. In this study, based on EAE chemical composition and antioxidant activities, we thought of using EAE and its isolated compounds as skin-care products.

A transdermal diffusion study of 5% EAE-cream formulation was conducted using the Franz diffusion cell and the start-M membrane. This technique of diffusion is similar to the dermal absorption of penetrants. The receptor compartment of the Franz cell showed a high transmission level of FM2, followed by FC. Our results correlate well with those found by Chuang et al. [50], which demonstrate the easy diffusion of flavonol glycosides through the membrane barrier. Therefore, FM2 and FC may easily penetrate the dermal barrier.

In favor of transdermal diffusion results, EAE, FM2, and FC were evaluated against two skin conditions, namely skin elasticity and skin pigmentation. 

In the epidermis, elastic fibers, consisting of fibrous protein elastin, are primarily associated with collagen fibers. Elastase is a key enzyme in the breakdown of connective tissue matrix proteins, such as elastin and collagen. Secretion and activation of this enzyme occurred as a result of dermal exposure to UV radiation and ROS [51]. Consequently, delaying or inhibiting elastase activity through natural antioxidant compounds could be a solution to protect the skin from elasticity problems such as wrinkles. To the best of our findings, EAE extract showed a high-glycoside flavonol composition, including quercetin-3-*O*-rhamnoside and myricetin-3-*O*-rhamnoside, which significantly contribute to its antioxidant activities. Moreover, this extract showed significant elastase inhibitory activity compared to the standard Epigallocatechin gallate. Additionally, FM2 and FC molecules inhibit elastase activity in a dose depending manner. Therefore, EAE and its FC and FM2 molecules could be used to treat skin elasticity issues caused by skin aging, wrinkles, or even wounds and burns [52].

The other skin condition assessed in this study is skin hyperpigmentation that appeared as brown spots, resulting from skin exposition to UV radiations or skin aging. This problem is caused by the over biosynthesis of melanin from the phenolic amino acid l-Tyrosine through a cascade of biochemical reactions. Three enzymes namely Tyrosinase, Tyrosinase Related Proteins-1 (TRP-1), and Tyrosinase Related Proteins-2 (TRP-2) are responsible for controlling this cascade of biochemical reactions [53]. As reported by Lee et al. [54], polyphenols and flavonoids have a high potential not only to inhibit tyrosinase, the key enzyme in melanin biosynthesis [1], but also TRP-1, TRP-2, and some toxic and mutagenic compounds [55]. In this study, pure molecules FC and FM2 showed an important tyrosinase inhibitory activity which is close to that of the standard Kojic acid, one of the most abundant inhibitors used against browning reactions in cosmetics [56]. This result agrees with that found by Kishore et al. [57], who mentioned that the flavonol glycosides have an important anti-tyrosinase activity. Besides, the cellular tyrosinase activity on B16 cells was evaluated. Pure molecules FC and FM2 exhibit higher cellular tyrosinase inhibition activity than EAE and Kojic acid. This finding is similar to the one found by Eghbali-Feriz et al. [58] and Tu and Tawata [59].

The cytotoxic effect on B16 melanoma cells is a result of stopping B16 cell proliferation by blocking cells during the G0/G1 phase of the cell cycle [60]. Interestingly, by comparing the IC_50_ values of the cellular tyrosinase activity with those of the anti-proliferative activity, EAE, FC, and FM2 are considered not cytotoxic on B16 cells to inhibit tyrosinase enzyme. Therefore, EAE, FC, and FM2 can be safely used as natural skin whitening agents and may replace the use of the standard kojic acid.

## 4. Materials and Methods

### 4.1. Chemicals and Reagents

The analytical grade solvents were bought from Carlo Erba-SDS (Val de Reuil, France). The HPLC grade and MS grade solvents were purchased from Acros Organic, Fisher Scientific, Illkirch, France. Milli-Q water, used for HPLC analysis, was obtained by a Millipore water purification system (Millipore, MilliQ, Paris, France). The used standards and reagents are; MTT (3-(4,5-dimethylthiazol-2-yl)-2,5-diphenyltetrazoliumbromide), DPPH (2,2-phenyl-1-picrylhydrazyl), Folin–Ciocalteu, dimethyl sulfoxide (DMSO), triton-X, ascorbic acid, quercetin, kojic acid, Epigallocatechin gallate, and gallic acid were purchased from (Sigma-Aldrich, St. Louis, MO, USA). Tyrosinase and elastase enzymes and *N*-succinyl-Ala-Ala-Ala-*p*-nitroanilide and l-3,4-dihydroxyphenylalanine (l-DOPA) substrates were bought from (Sigma-Aldrich, St. Louis, MO, USA). Penicillin-streptomycin, fetal bovine serum (FBS), and Dulbecco’s modified eagle medium (DMEM) were purchased from Life Technologies (Gibco, Paisley, UK). Hydro-cream emulsion was purchased from Excipial formulae (Galderma, Alby-Sur-chéran, France). The strat-M membrane test model (25 mm disc size) (Ref SKBM02560) (Bedford, MA, USA) was used for the transdermal diffusion assay.

### 4.2. Plant Preparation

The leaves of *P. lentiscus* L. were collected in March from the Zaghouan region in the North-East of Tunisia (36°22′30.6″ North and 10°11′15.2″ East). The plant was identified by Maher Boukhris botanist in the Laboratory of Plant Biotechnologies Applied to Crop Improvement, Faculty of Sciences of Sfax, University of Sfax, Sfax, Tunisia. A voucher specimen of this plant was deposited in the laboratory under the number of LBP-PE 2017-3. Industrial-scale steam distillation [61] of the leaves was performed to extract the essential oil fraction. Then, the distilled leaves by-product was air-dried in the darkness, grounded into a fine powder, and kept at 4 °C until use.

### 4.3. Extracts Characterization

#### 4.3.1. PSE Procedure

150 g of PDL were subjected to PSE. It was performed using a Speed Extractor Büchi apparatus (E-914) (Fawil, Switzerland) and the methanol as the extracting solvent (200 mL total volume). The extraction step was composed of two 10 min cycles containing a 5 min heating time and a solvent pumping at 1500 psi and 50 °C. The ME (35 g) was dried under a vacuum. Then, it was separated into organic and aqueous fractions in a separating funnel vigorously shaken using EtOAc/H_2_O (1:1, *v/v*), and left to stand until the separation of the phases. They were then dried separately under a vacuum and stored at −4 °C for further analysis.

#### 4.3.2. Total Phenolic Content

TPC was spectrophotometrically evaluated using the Folin–Ciocalteu method described by Kumaran and Karunakaran [62]. Results were mentioned as mg Gallic Acid Equivalent (GAE) per g of extract.

#### 4.3.3. Total Flavonoid Content

The TFC was measured using the AlCl_3_ method described by Bahorun [63]. The flavonoid concentration was expressed as mg Quercetin Equivalent (QE) per g of extract.

#### 4.3.4. Antioxidant Activities

##### DPPH Radical Scavenging Assay

The DPPH scavenging test was carried out using the method mentioned by Brand-Williams et al. [64]. The percentage of DPPH scavenging was measured using Equation (1):

% DPPH scavenging = (OD_control_ − OD_sample_)/OD_control_ × 100 (1)

##### FRAP Assay

The FRAP was evaluated using the method described by Benzie and Strain [65]. The ascorbic acid standard was used as a control. The reducing iron power was expressed as mg Ascorbic Acid Equivalent (AEAC) per g of extract.

### 4.4. Identification of Phenolic Compounds

#### 4.4.1. HPLC-DAD and HPLC-MS Procedures

HPLC-DAD analyses were carried out using Thermo Scientific Dionex U3000 apparatus (Les Ulis, France), as described by Chin et al. [66] with certain modifications. The stationary phase used for compounds separation is a phenyl-hexyl column (X select CSH, 4.6 × 250 mm, 5 μm). The two systems of solvents used as a mobile phase are; (A) MeCN/H_2_O/HCOOH with a proportion of 50:49.95:0.05 (*v*/*v*/*v*) and (B) H_2_O/HCOOH with a proportion of 99.9:0.1 (*v*/*v*). The gradient of solvent was starting from 15% of (A) and increasing to 72% during 15 min, then isocratic at 72% of (A) during 13 min, from 72 to 30 of (A) during 5 min, from 30 to 20% of (A) during 2 min, and finally from 20 to 15% of (A) during 10 min. The volume of sample injection was 20 µL and the flow rate was 0.7 mL/min. The chromatograms of phenolic compounds were recorded at 280 nm.

The same method of separation was operated for HPLC-MS analyses. The mass spectrum was acquired in the negative ionization mode using the following operating conditions: ionic excitation voltage 3 kV, cone voltage 50 V, Q energy 70 V, and desolvation temperature 500 °C. The *m/z* was ranged from 100 to 1000. Chromeleon^®^ software ^®^, version 6.8, provided by Thermo Scientific Dionex (Les Ulis, France) was used for the treatment of results.

#### 4.4.2. CPC Separation

Different systems of solvents from the ARIZONA scale of polarity were tested for the CPC separation. The solvents system was selected according to an estimation of the partition coefficient K, defined as the ratio of concentrations of the compounds between the two non-miscible phases. K was evaluated by the shake-flask method performed by adding 2 mg of extract to the previously equilibrated biphasic solvents systems (4 mL) in test vials. The vials were vigorously shaken. After decantation, TLC examination was carried out under UV light (at 254 nm and 366 nm) and after spraying with a solution of sulfuric vanillin. This test has led to choosing the solvents system composed of c-Hex/EtOAc/MeOH/H_2_O (1:2:1:2, *v/v/v/v*) (ARIZONA system K) for the CPC separation.

The CPC separation was carried out with an SCPC-250 + 1000B apparatus (Armen Instrument, Sain-Avé, France). The stationary and mobile phases are respectively the lower and upper phases of the prepared biphasic solvents system. The CPC was carried out in ascending mode using the 1000 mL rotor, a flow rate of 20 mL/min, and a rotation speed of 1000 rpm. The sample was firstly dissolved in the upper phase of the biphasic solvent system and then injected into the apparatus. An extrusion step was performed using the stationary phase as a mobile phase. The eluted fractions were analyzed using TLC monitored with EtOAc/MeOH (7:2, *v/v*).

#### 4.4.3. MPLC Separation

The stationary phase of separation was composed of a column Flash vide (AIT-France type) filled with a silica gel LC60A 20–45 M type. The mobile phase composed of DCM/MeOH (95:5, *v/v*) was pumped through the column using Büchi pump manager C-615 at a flow of 5 mL/min. The eluted fractions were collected by automatic Büchi fraction collector C-660. TLC analysis of fractions was monitored with EtOAc/MeOH (7:2, *v/v*).

#### 4.4.4. Preparative HPLC

Preparative HPLC separation was carried out using Armen Instrument (Saint-Avé, France) equipped with a Büchi filter-photometer detector, a Büchi C-660 collector, and a PFP column (250 × 32.5 µm × 5 µm). The detector parameters are; integrator MTT = 7, range detector = 0.64, constant time = 1. Samples were dissolved in methanol and filtered using an Acrodisc© Premium 25 mm. Two methods were used. Method E1: the solvent gradient was started with MeOH/H_2_O at 20:80, *v*/*v* for 8 min then changed to 100:0, *v*/*v* for 68 min. Method E2: the solvent gradient was started with MeOH/H_2_O at 40:60, *v*/*v* for 12 min then, increased to 100:0, *v*/*v* for 51 min.

#### 4.4.5. Determination of Isolated Compounds Structure

The chemical structures of isolated compounds were determined by combining NMR data in 1D and 2D (COSY, HSQC, and HMBC) and MS data. NMR spectra of samples dissolved in MeOD-*d*_4_ were acquired by Bruker 400 MHz spectrometer (Wissembourg, France) using standard Varian pulse sequences. Mass spectrometer ZQ 2000 Waters (Saint Quentin, France) was used to determine the mass of our compounds. The mass spectrum was recorded in the negative ionization mode using a collision cell RF of 500.0 Vpp, a capillary voltage of 4.0 kV, a nebulizer of 7.25 psi, and a dry heater of 200 °C. The acquired scan ranges from *m/z* 50 to 3000.

#### 4.4.6. Validation of HPLC-DAD Method and Quantification of Purified Molecules

The HPLC-DAD method was validated for quantification of the isolated molecules, according to Fernandes et al. [67], where the used standards are the pure molecule isolated from the extract. For this aim, external calibration curves with three levels of concentrations (low, medium, and high; from 0.05 to 0.7 mg/mL prepared with methanol) were plotted for each isolated compound. The linear regression equation and the linear correlation coefficient (r^2^) of each compound were determined. Each concentration solution of each compound was analyzed three times a day and for three days. The precision of the method was expressed as RSD%.

Additionally, EAE was analyzed at a concentration of 10 mg/mL with five replicates injections. It was also analyzed after adding each isolated compound at three concentration levels. The accuracy percentage of the method was calculated [67]. Limit of detection (LOD) and limit of quantification (LOQ) were also determined. They were calculated from the three analytical curves as follows: LOQ = 10 σ/S and LOD = 3.3 σ/S, in which S is the slope and σ is the standard deviation of the intercept.

PDL content of isolated compounds was expressed as mg of pure molecule/g of dry weight. 

### 4.5. Transdermal Diffusion Test

Transdermal diffusion was evaluated using a Franz diffusion cell system, as mentioned by Chuang et al. [50] with certain modifications. Firstly, the start-M membrane, a model membrane for transdermal diffusion, was mounted in a Franz diffusion cell. Then, 5 mL of 0.1 M sodium phosphate buffer (PBS) and 1 g of extract formulation cream were placed in the receptor and the donor compartments, respectively. The extract formulation cream was prepared by mixing the hydro-cream base emulsion purchased from Galderma and the EAE at 5% (*w*/*w*) [68,69] by a mechanical stirrer (VMI Rayneri, Francelab, Paris, France) [70]. The receptor medium of the Franz cell was maintained at 37 °C and stirred with a magnetic stirrer at 400 rpm. After 24 h, the receptor medium was recovered, evaporated, freeze-dried, and then 4 mL of MeOH was added. The sample was filtered through a 0.45 filter and analyzed by HPLC-MS to determine the bioactive molecules that were released from the start-M membrane. The bioactive molecules that crossed the start-M membrane were then quantified, as described in Section 4.4.6, by injecting the receptor medium through HPLC-DAD at a concentration of 10 mg/mL. Results were expressed as µg of molecule crossed the membrane/g of 5% EAE-hydro-cream.

### 4.6. In Vitro Enzymatic Activities

#### 4.6.1. Tyrosinase Inhibition

The inhibitory effect of our samples on the mushroom tyrosinase enzyme was analyzed using the substrate l-DOPA [71]. The extract and the pure molecules were evaluated at concentrations ranging between 12 and 200 µg/mL. The positive control of kojic acid was prepared in the same conditions. The tyrosinase inhibition was measured as follows:

% Tyrosinase inhibition *=* (OD_control_ − OD_sample_)/OD_control_ × 100 (2)

#### 4.6.2. Elastase Inhibition

The elastase inhibitory effect of our samples was evaluated as described by Angelis et al. [72], using the enzyme porcine pancreatic elastase and the substrate *N*-succinyl-Ala-Ala-Ala-*p*-nitroanilide. The extract and the pure molecules were evaluated at concentrations ranging between 12 and 100 µg/mL. Epigallocatechin gallate was used as a positive control. The elastase inhibition was measured using Equation (3):

% Elastase inhibition = (OD_control_ − OD_sample_) OD_control_ × 100 (3)

### 4.7. Cell Culture Conditions

B16 cells (C57BL/6, Resource No. RBRC-RCB0557) used in this study were prepared as described by Elloumi et al. [73].

#### 4.7.1. Cell Viability Analysis

The MTT assay was performed to evaluate the cytotoxicity effect of our samples on B16 cells [74]. Different sample concentrations (2.5–80 µg/mL) were applied to the Cells. The cellular tyrosinase inhibition was measured as follows:

% Cell viability = (OD_sample_/OD_control_) × 100 (4)

#### 4.7.2. Cellular Tyrosinase Activity Assay

The cellular tyrosinase activity on B16 cells was evaluated as described by Kamei et al. [75] with some modifications. Briefly, B16 cells were incubated in a 96-well plate (25 × 10^3^ cells/well) for 24 h. Then, they were treated with our samples and the positive control kojic acid. After that, the cells were washed with PBS and lysed with PBS supplemented with 1% of Triton-X/PBS, after 48 h of samples exposure. A volume of 100 µL of 0.1% l-DOPA was added to the wells for 1 h. The absorbance was mesuread at 492 nm. The cellular tyrosinase inhibition was measured as follows:

% Cellular tyrosinase inhibition = (OD_control_ − OD_sample_)/OD_control_ × 100 (5)

### 4.8. Statistical Analysis

The results were mentioned as mean ± standard deviation. Statistical significance was analyzed using GraphPad Prism 6. Means values were compared with the ANOVA test. *p*-value < 0.001 and *p*-value < 0.0001 were considered statistically significant.

## 5. Conclusions

The present study revealed that the EAE of PDL contains a rich flavonol glycoside composition and represents a good antioxidant potential. It has been fractionated using several chromatographic techniques. Three main molecules were isolated and then quantified, which are FC, FM2, and FB2. The transdermal diffusion study revealed that the FM2 molecule was transmitted with a high amount through the membrane barrier, followed by the FC molecule. Consequently, EAE, FC, and FM2 are suggested to be good skin-care products. The tyrosinase and elastase inhibition assays and the intracellular tyrosinase inhibition on B16 cells showed that EAE, FC, and FM2 have important inhibitory activities compared to the well-known standards, at non-cytotoxic concentrations. Therefore, EAE, FC, and FM2 can be excellent natural products for the treatment of hyperpigmentation and skin elasticity conditions, resulting mainly from skin aging.

## Figures and Tables

**Table 1:** Total phenolic and flavonoid contents of the methanol extract (ME) of *P. lentiscus* L. distilled leaves and its ethyl acetate (EAE) and aqueous (AE) fractions.

Extracts	Total Phenolic (mg GAE/g of Extract)	Total Flavonoids (mg QE/g of Extract)
ME	156.43 ± 1.08	3.52 ± 1.00
EAE	152.92 ± 0.05	3.48 ± 0.58
AE	148.11 ± 0.05 *	2.30 ± 0.56 **

Mean ± SD (*n* = 3). * significant *p* ˂ 0.001 compared to EAE. ** significant *p* ˂ 0.0001 compared to EAE. GAE = gallic acid equivalent. QE = quercetin equivalent.

**Table 2:** The antioxidant activities of the methanol extract (ME) of *P. lentiscus* L. distilled leaves and its ethyl acetate (EAE) and aqueous (AE) extracts were determined using DPPH radical scavenging and FRAP assays.

Extracts	DPPH IC_50_ (µg/mL)	FRAP (mg AEAC/g of Extract)
ME	19.62	467.29 ± 21.77 **
EAE	18.07	522.76 ± 22.99
AE	19.52	421.91 ± 15.48 **
Ascorbic acid	13.85	-

Mean ± SD (*n* = 3). ** significant *p* ˂ 0.0001 compared to EAE. DPPH = 2,2-phenyl-1-picrylhydrazyl. FRAP = Ferric reducing antioxidant power. Ascorbic acid is a standard. AEAC = ascorbic acid equivalent.

**Table 3:** Peaks assignment of ethyl acetate extract (EAE) of *P. lentiscus* L. distilled leaves, acquired by HPLC-MS (peak numbers have been recorded in Figure 1).

Peak No.	Rt (min)	[M − H]^−^ (*m*/*z*)	λ_max_ (nm)	Identification
2	8.70	479	260/355	Myricetin-3-*O*-galactoside
3	11.23	441	217/276	Epicatechin Gallate
7	17.88	495	219/276	Digalloylquinic Acid
8	18.87	495	217/278	Digalloylquinic Acid
14	22.71	479	260/350	Myricetin-3-*O*-hexoside
15	23.1	447	260/355	Kaempferol-3-*O*-glucoside
16	23.69	463	263/350	Myricetin-3-*O*-rhamnoside
17	24.59	463	269/351	Quercetin-3-*O*-galactoside
19	25.74	465	280/340	Taxifolin 3′-*O*-glucoside
20	26.5	447	258/351	Quercetin-3-*O*-rhamnoside
21	27.42	447	272/345	Quercetin-*O*-deoxyhexoside
23	29.15	615	267/354	Myricetin-*O*-galloyl-deoxyhexoside
24	29.64	481	280/350	Ampelopsin-*O*-hexoside
26	31.85	431	267/348	Kaempferol-3-*O*-rhamnoside

**Table 4:** Quantification of quercetin-3-*O*-rhamnoside (FC), myricetin-3-*O*-rhamnoside (FM2), and kaempferol-3-*O*-rhamnoside (FB2), present in *P. lentiscus* L. distilled leaves (PDL) and after crossing the start-M membrane (24 h of incubation).

**Compounds**	**Linear Regression Equation**	**LOQ (mg/mL)**	**LOD (mg/mL)**	**r** **^2^**	**Repeatability (% RSD)**		
					**Low Level**	**Medium Level**	**High Level**
FC	Y = 143.1X + 1.24	0.311	0.102	0.993	2.039	0.936	1.920
FM2	Y = 165.2X + 1.30	0.029	0.009	0.997	3.150	2.190	2.241
FB2	Y = 102.5X + 0.19	0.026	0.008	0.998	3.950	0.645	0.275
	**Intermediate Precision (% RSD)**				**Accuracy %**		
	**Low Level**	**Medium Level**	**High Level**		**Low Level**	**Medium Level**	**High Level**
FC	1.656	0.995	0.712		100.932	96.174	102.950
FM2	1.829	1.805	0.907		115.927	115.927	110.60
FB2	1.630	0.922	0.321		123.449	123.449	99.651
	**mg of Pure Molecule/g Dry Weight**				**µg of Crossed Molecule/g of 5%** **Hydro-cream**		
FC	10.471 ± 0.261				3.161 ± 0.006		
FM2	12.173 ± 0.742				52.102 ± 0.001		
FB2	4.530 ± 0.592				-		

Limit of detection (LOD), limit of quantification (LOQ), precision (RSD%), and accuracy (%) were calculated for validation of the HPLC method. Mean ± SD (*n* = 9).

## Data Availability

All the data that support the findings of this study are available on request from the corresponding author.

## Supplementary Materials

The following are available online, Figure S1. ^1^H-NMR (400 MHz, MeOD-*d*_4_) spectrum of quercetin-3-*O*-rhamnoside. Figure S2. COSY ^1^H-^1^H-NMR (400 MHz, MeOD-*d*_4_) spectrum of quercetin-3-*O*-rhamnoside. Figure S3. HSQC NMR (400 MHz, MeOD-*d*_4_) spectrum of quercetin-3-*O*-rhamnoside. Figure S4. HMBC NMR (400 MHz, MeOD-*d*_4_) spectrum of quercetin-3-*O*-rhamnoside. Figure S5. MS (ESI^−^) spectra of quercetin-3-*O*-rhamnoside. Figure S6. ^1^H-NMR (400 MHz, MeOD-*d*_4_) spectrum of lupeol. Figure S7. COSY ^1^H-^1^H-NMR (400 MHz, MeOD-*d*_4_) spectrum of lupeol. Figure S8. HSQC NMR (400 MHz, MeOD-*d*_4_) spectrum of lupeol. Figure S9. HMBC NMR (400 MHz, MeOD-*d*_4_) spectrum of lupeol. Figure S10. MS (ESI^−^) spectrum of lupeol. Figure S11. ^1^H-NMR (400 MHz, MeOD-*d*_4_) spectrum of loliolide. Figure S12. COSY ^1^H-^1^H-NMR (400 MHz, MeOD-*d*_4_) spectrum of loliolide. Figure S13. MS (ESI^−^) spectrum of loliolide. Figure S14. ^1^H-NMR (400 MHz, MeOD-*d*_4_) spectrum of myricetin-3-*O*-rhamnoside. Figure S15. COSY ^1^H-^1^H-NMR (400 MHz, MeOD-*d*_4_) spectrum of myricetin-3-*O*-rhamnoside. Figure S16. MS (ESI^−^) spectrum of myricetin-3-*O*-rhamnoside. Figure S17. ^1^H-NMR (400 MHz, MeOD-*d*_4_) spectrum of kaempferol-3-*O*-rhamnoside. Figure S18. COSY ^1^H-^1^H-NMR (400 MHz, MeOD-*d*_4_) spectrum of kaempferol-3-*O*-rhamnoside. Figure S19. HSQC NMR (400 MHz, MeOD-*d*_4_) spectrum of kaempferol-3-*O*-rhamnoside. Figure S20. HMBC NMR (400 MHz, MeOD-*d*_4_) spectrum of kaempferol-3-*O*-rhamnoside. Figure S21. MS (ESI^−^) spectrum of kaempferol-3-*O*-rhamnoside. Table S1. NMR data of flavonol glycosides (400 MHz, MeOD-*d*_4_) (position numbers have been recorded in Figure 4). Table S2. ^1^H-NMR data of loliolide and lupeol (400 MHz, MeOD-*d*_4_) (position numbers have been recorded in Figure 4).

## Sample Availability

Samples of the compounds quercetin-3-*O*-rhamnoside, myricetin-3-*O*-rhamnoside, and kaempferol-3-*O*-rhamnoside used during the current study are available from the corresponding author on reasonable request.
