# Supplementary material for: Pistacia lentiscus L. Distilled Leaves as a Potential Cosmeceutical Ingredient: Phytochemical Characterization, Transdermal Diffusion, and Anti-Elastase and Anti-Tyrosinase Activities
Source: Molecules. 2022 Jan 27;27(3):855. doi: 10.3390/molecules27030855 (PMC8838631; doi:10.3390/molecules27030855)
Supplement: Supplementary file 1 [file molecules-27-00855-s001.zip › molecules-1516108-supplementary.pdf]

Supplementary Material

Pistacia lentiscus L. Distilled Leaves as a Potential Cosmeceutical Ingredient: Phytochemical Characterization, Transdermal Diffusion, and Anti-elastase and Anti-tyrosinase Activities

Wiem Elloumi [1], Amina Maalej [1], Sergio Ortiz [2], Sylvie Michel [2], Mohamed Chamkha [1], Sabrina Boutefnouchet [2] and Sami Sayadi [3,*]

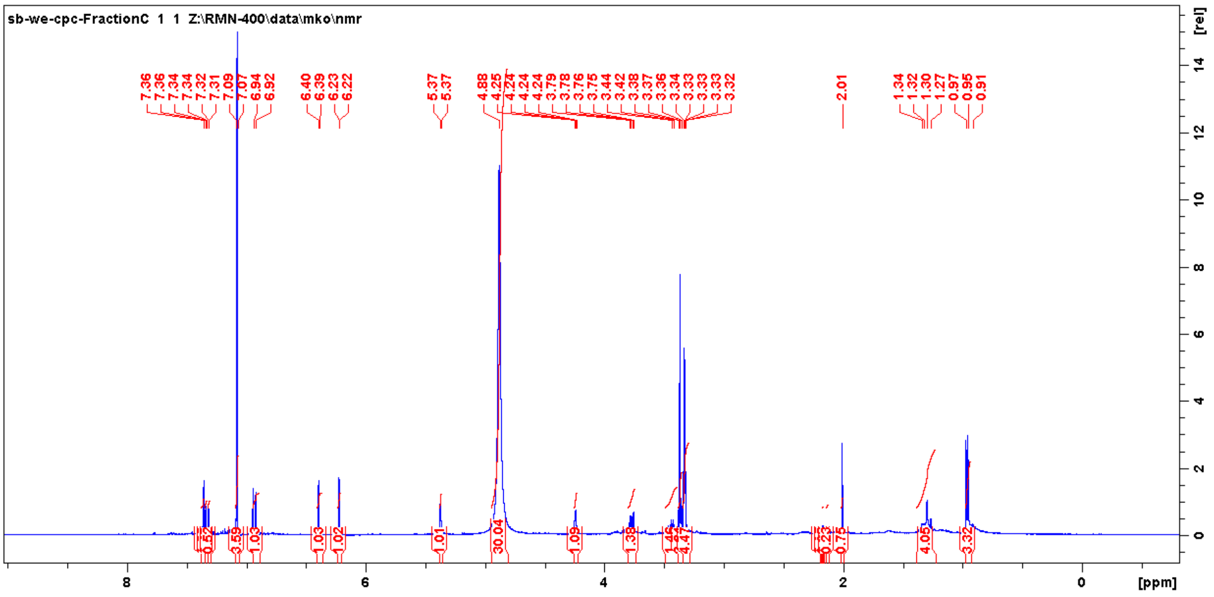

Figure S1. ^{1}H-NMR (400 MHz, MeOD-*d*$_4$) spectrum of quercetin-3-*O*-rhamnoside.

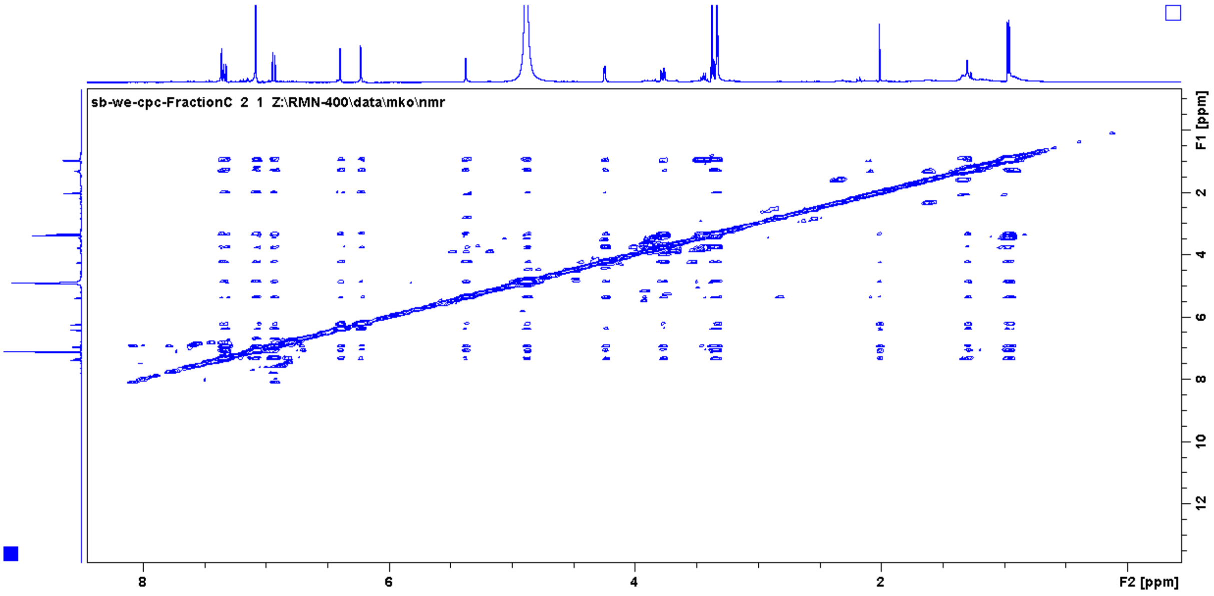

Figure S2. COSY ^{1}H-^{1}H-NMR (400 MHz, MeOD-*d*$_4$) spectrum of quercetin-3-*O*-rhamnoside.

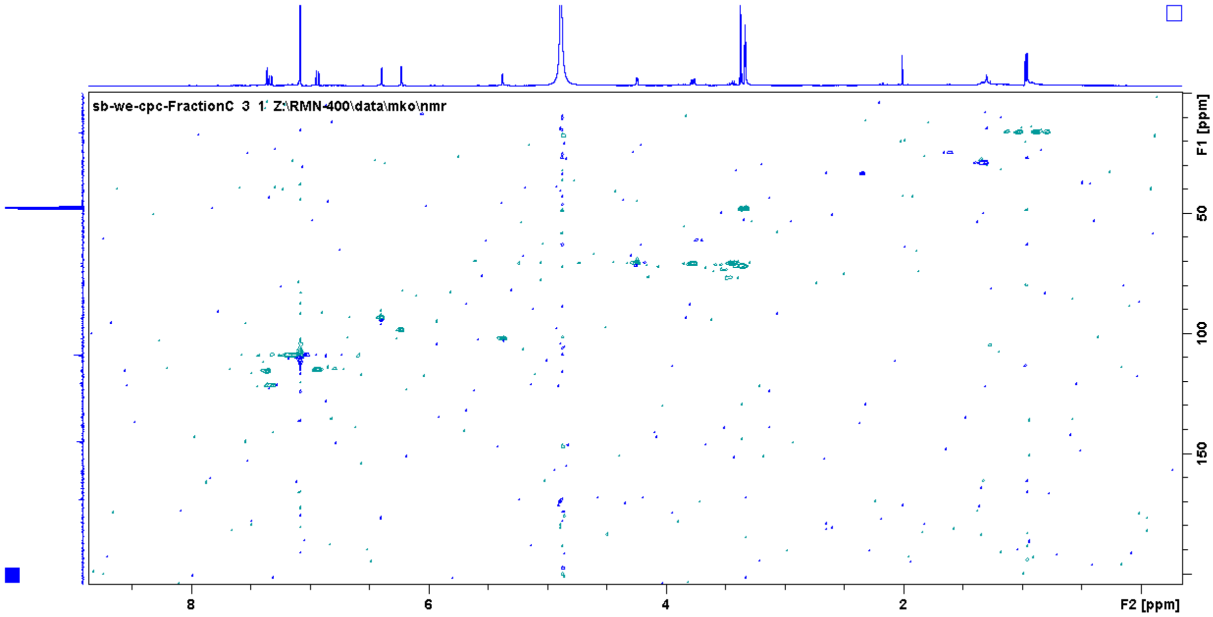

Figure S3. HSQC NMR (400 MHz, MeOD-d_4) spectrum of quercetin-3-*O*-rhamnoside.

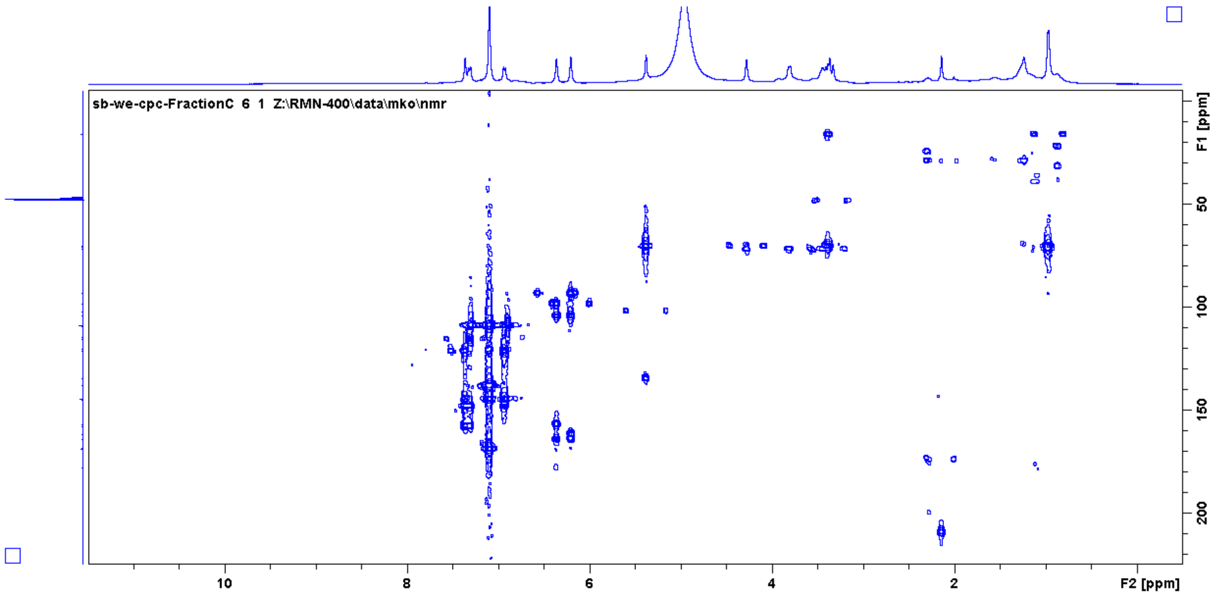

Figure S4. HMBC NMR (400 MHz, MeOD-d_4) spectrum of quercetin-3-*O*-rhamnoside.

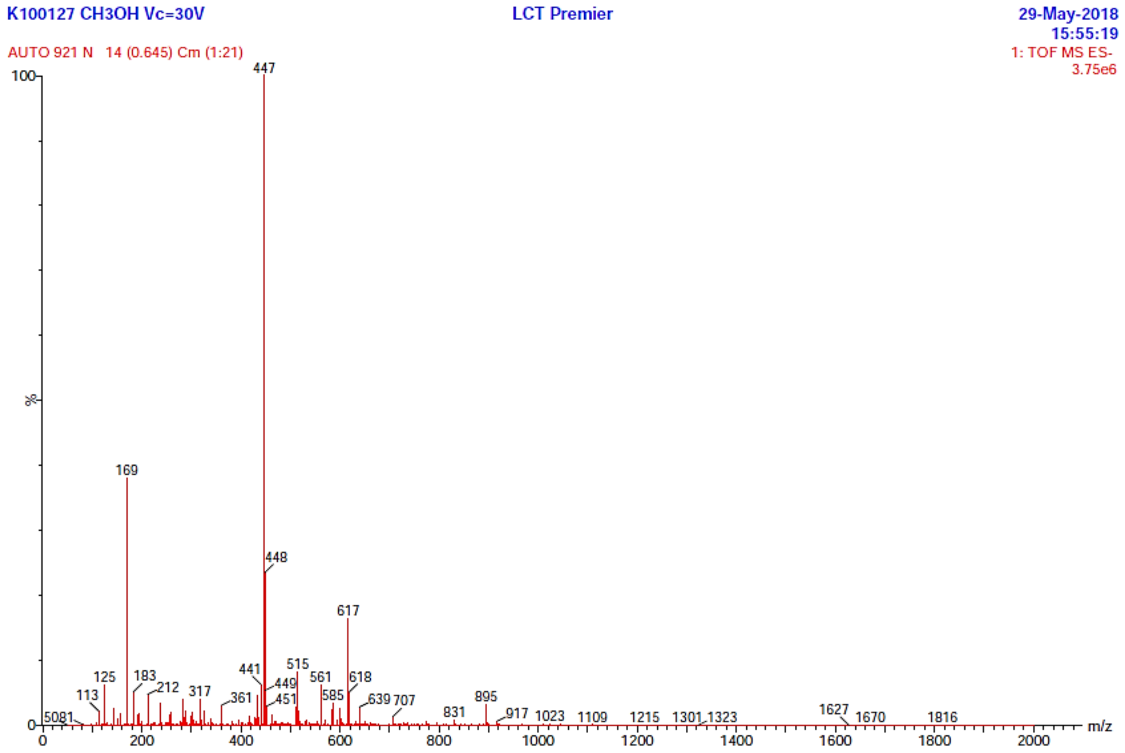

Figure S5. MS (ESI-) spectra of quercetin-3-*O*-rhamnoside.

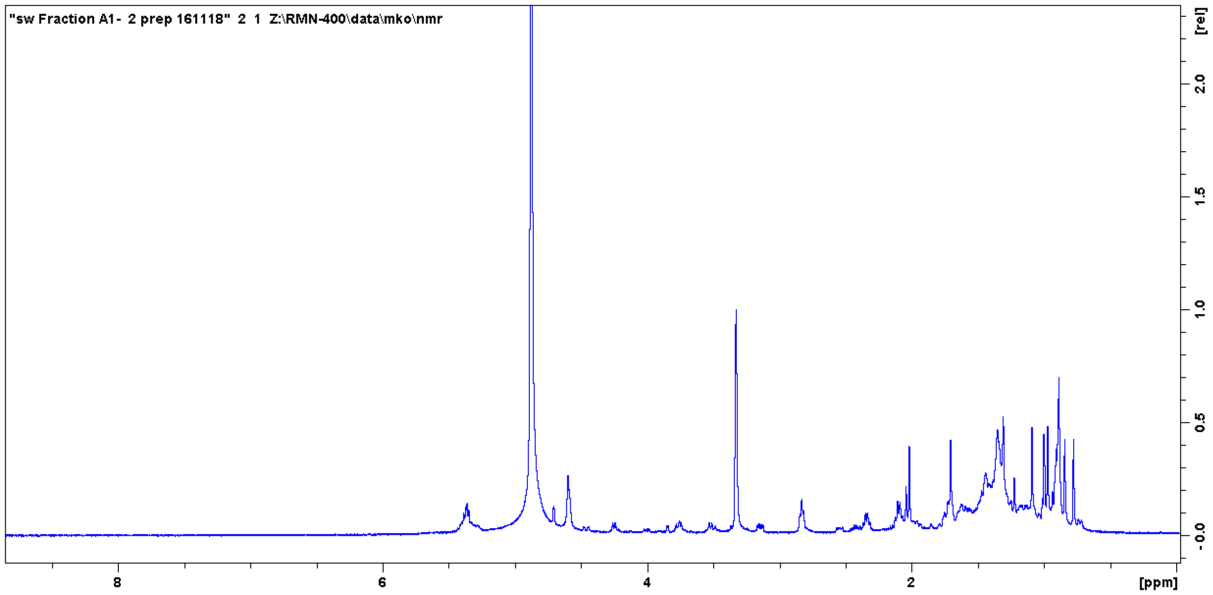

Figure S6. ^{1}H-NMR (400 MHz, MeOD-d_4) spectrum of lupeol.

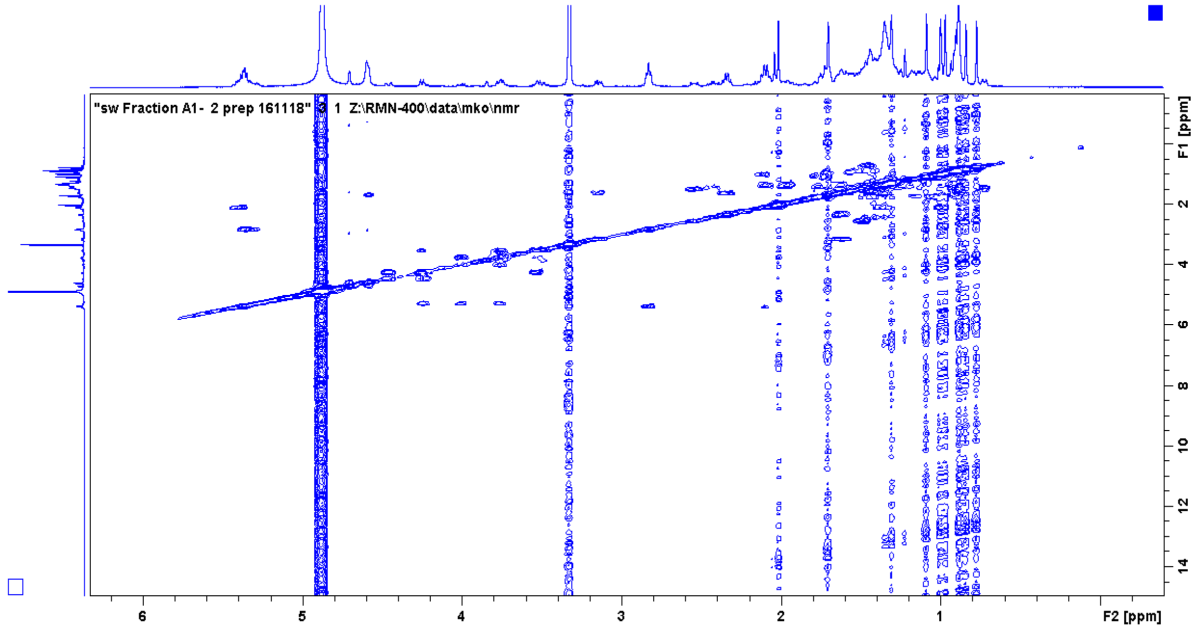

Figure S7. COSY ^1H-^1H-NMR (400 MHz, MeOD-*d*$_4$) spectrum of lupeol.

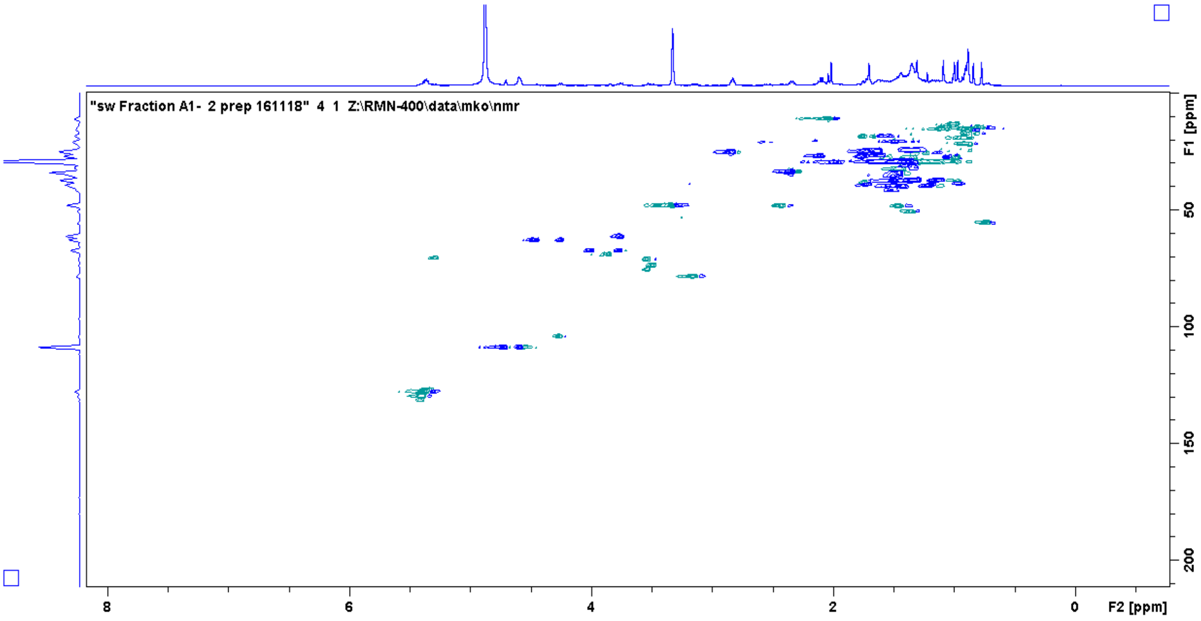

Figure S8. HSQC NMR (400 MHz, MeOD-*d*$_4$) spectrum of lupeol.

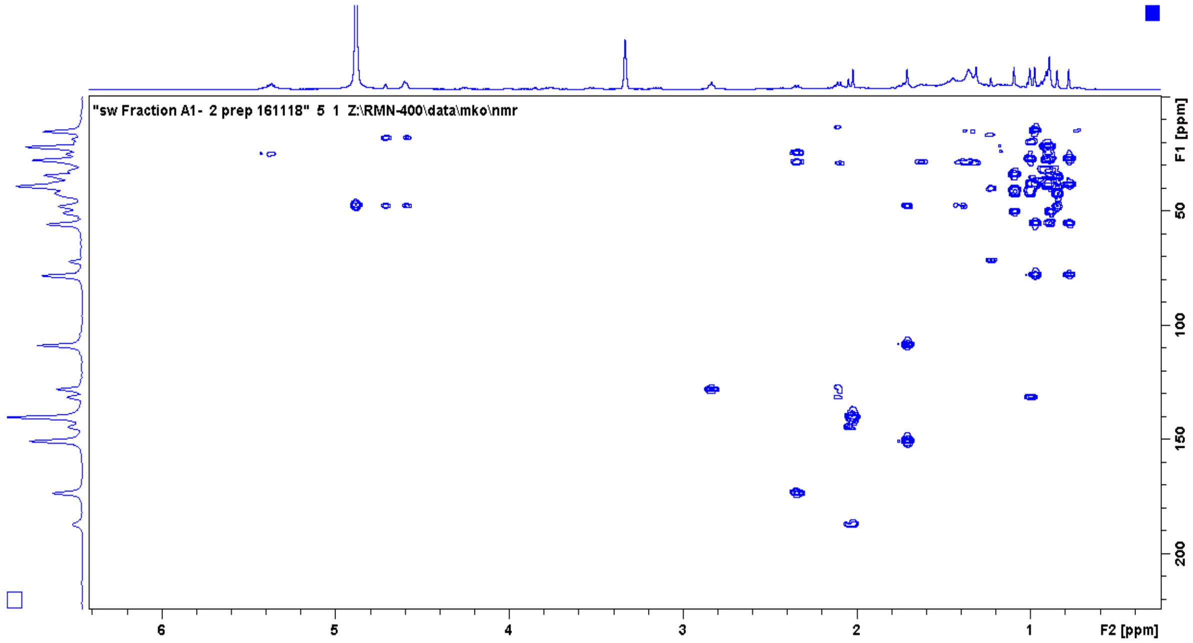

Figure S9. HMBC NMR (400 MHz, MeOD-d_4) spectrum of lupeol.

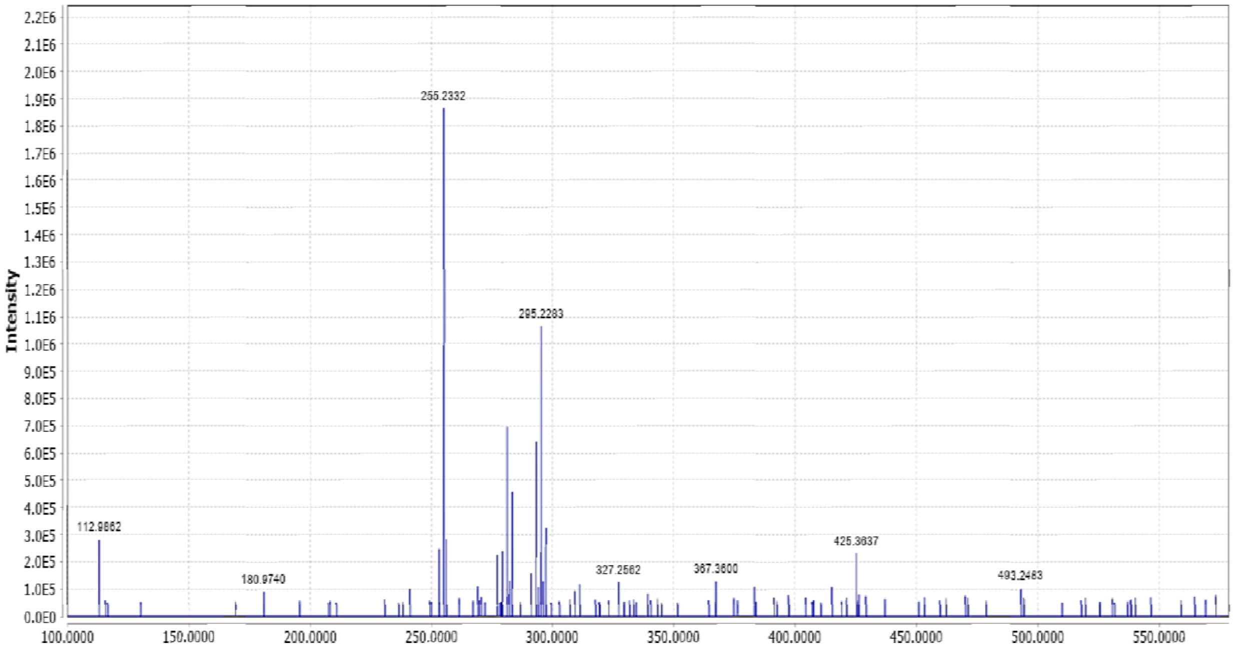

Figure S10. MS (ESI$^-$) spectrum of lupeol.

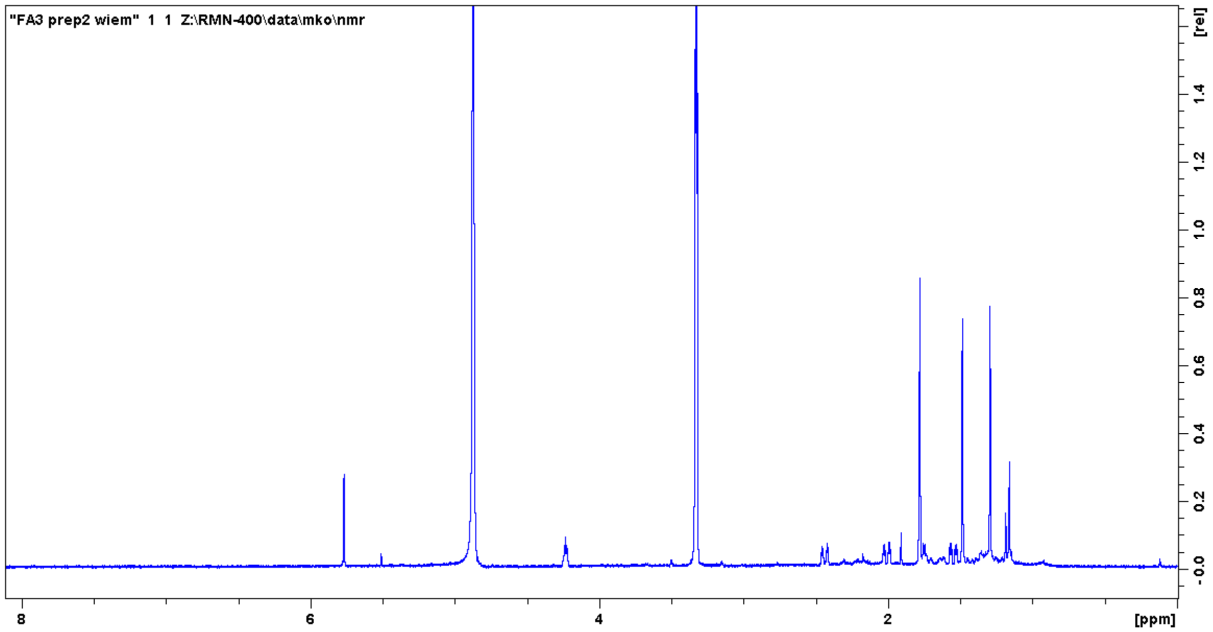

Figure S11. ^{1}H-NMR (400 MHz, MeOD-d_4) spectrum of loliolide.

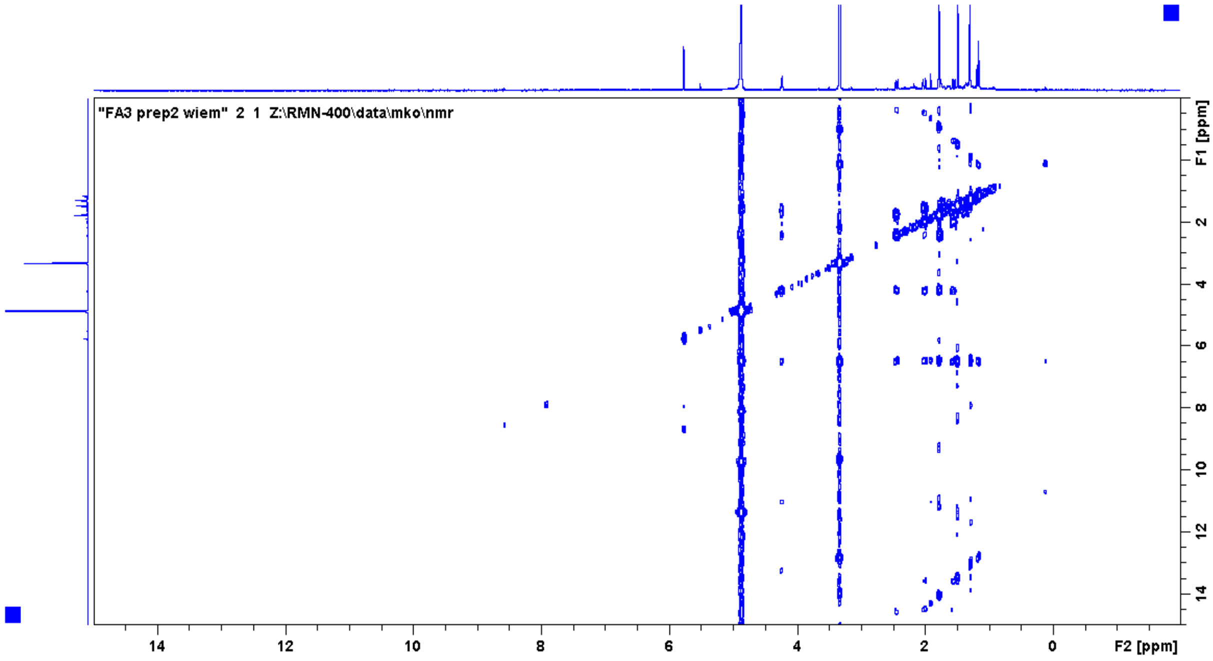

Figure S12. COSY ^{1}H-^{1}H-NMR (400 MHz, MeOD-d_4) spectrum of loliolide.

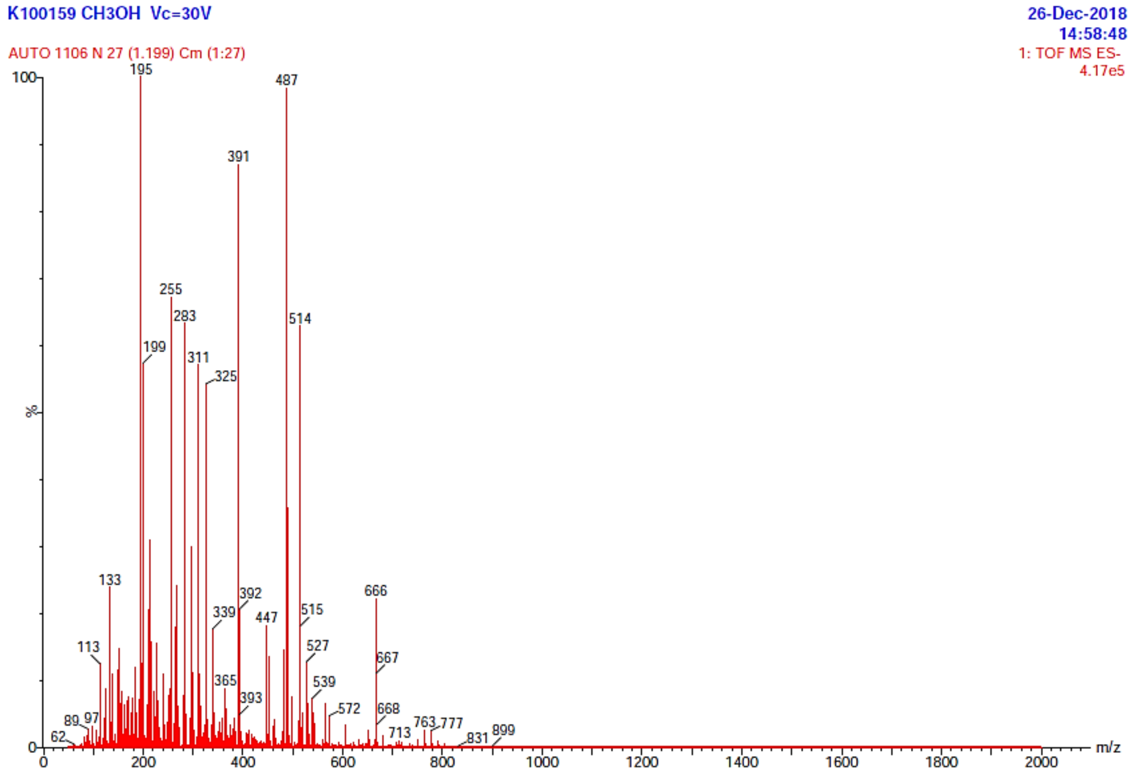

Figure S13. MS (ESI$^-$) spectrum of loliolide.

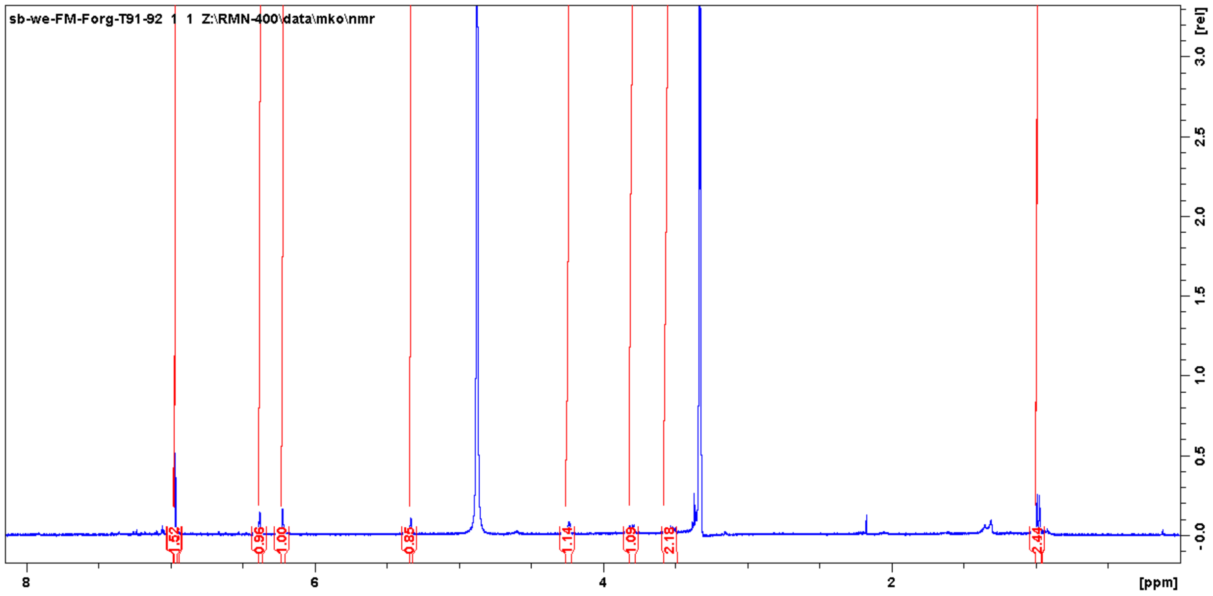

Figure S14. ^{1}H-NMR (400 MHz, MeOD-d_4) spectrum of myricetin-3-*O*-rhamnoside.

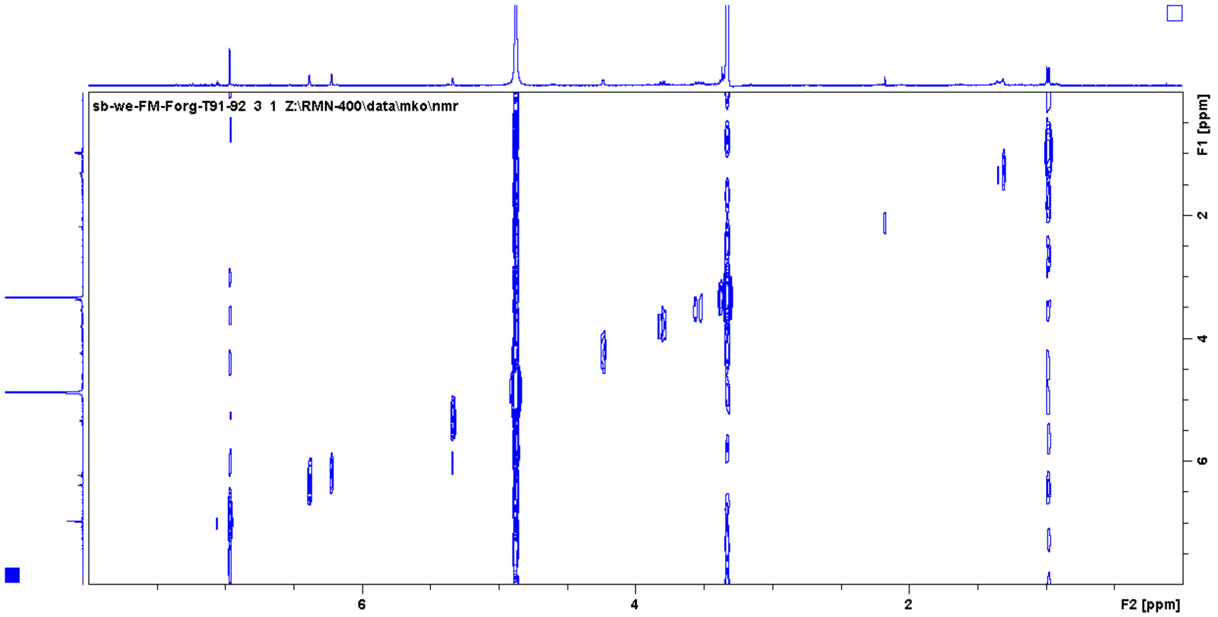

Figure S15. COSY ^{1}H-^{1}H-NMR (400 MHz, MeOD-d_4) spectrum of myricetin-3-*O*-rhamnoside.

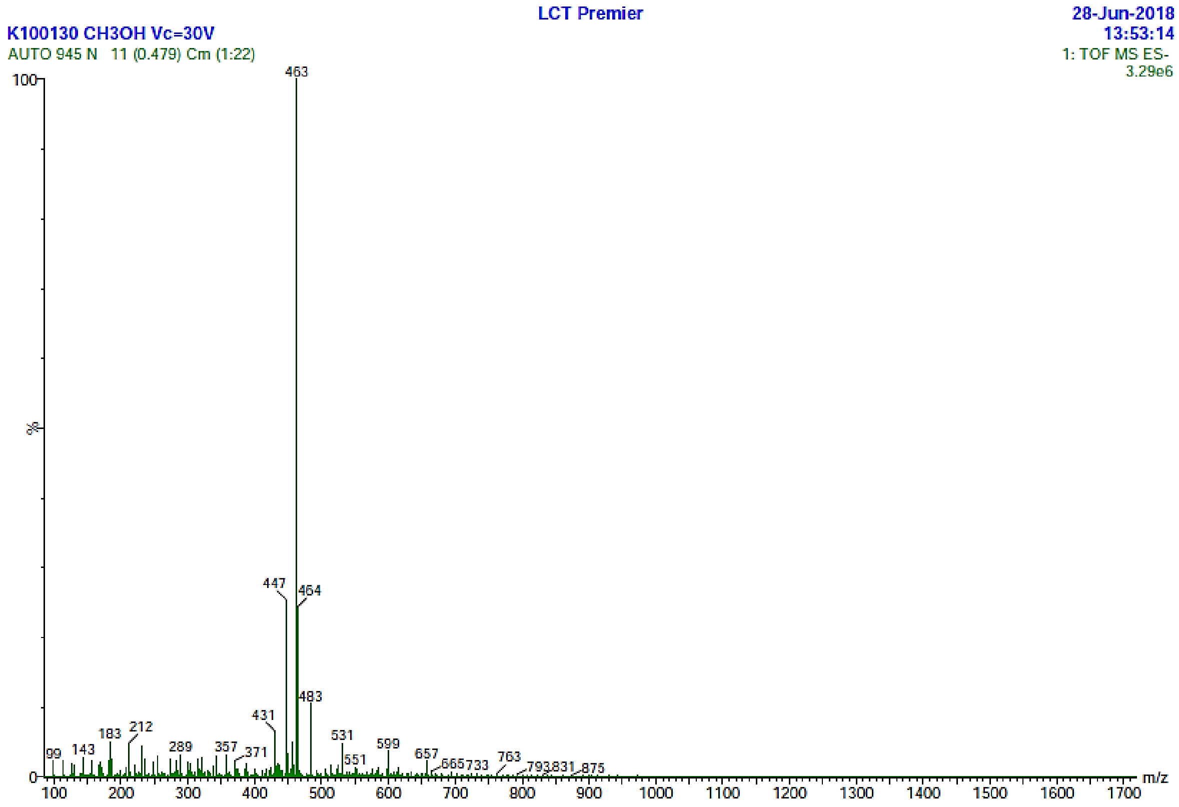

Figure S16. MS (ESI^{-}) spectrum of myricetin-3-*O*-rhamnoside.

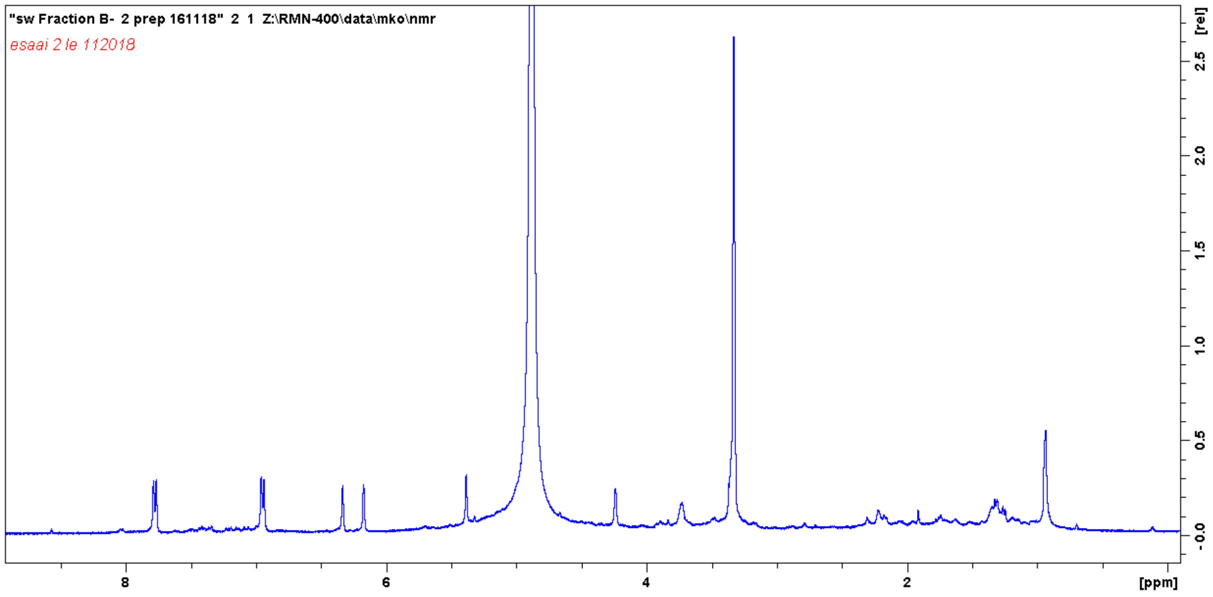

Figure S17. ¹H-NMR (400 MHz, MeOD-d_4) spectrum of kaempferol-3-*O*-rhamnoside.

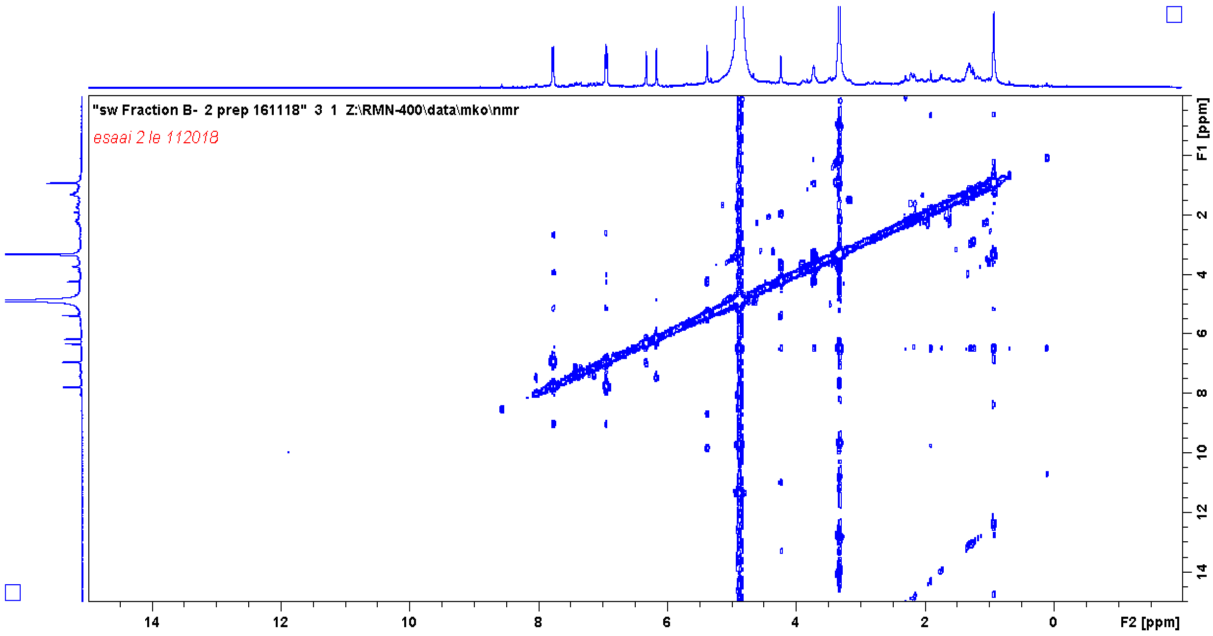

Figure S18. COSY ¹H-¹H-NMR (400 MHz, MeOD-d_4) spectrum of kaempferol-3-*O*-rhamnoside.

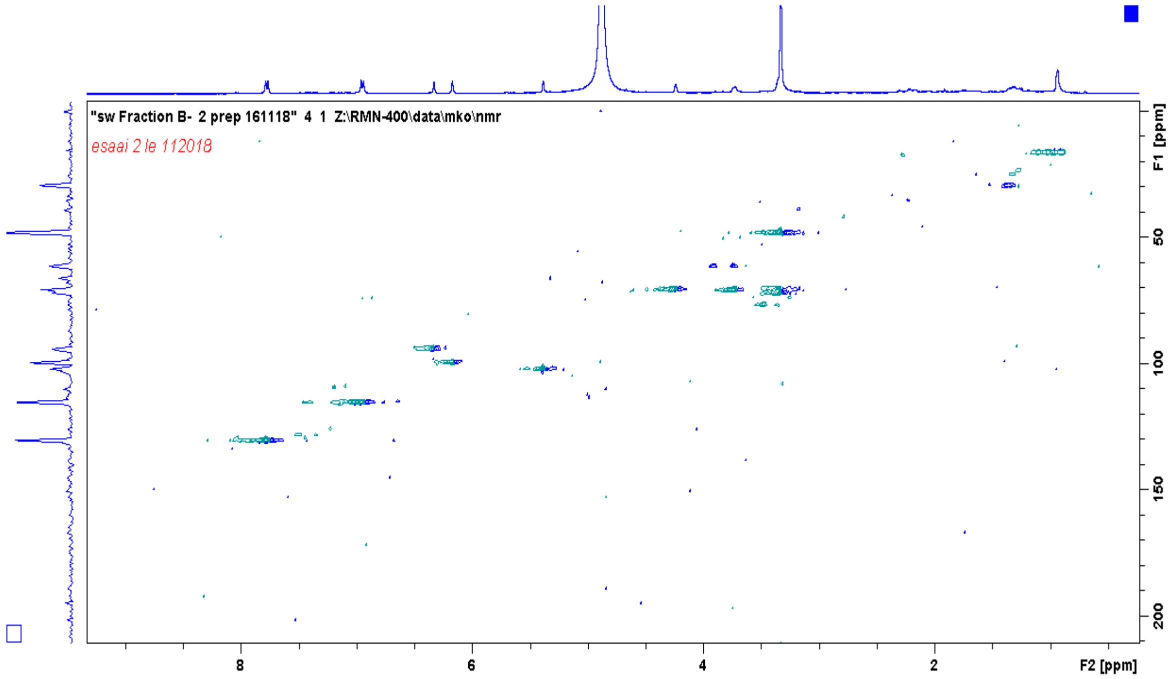

Figure S19. HSQC NMR (400 MHz, MeOD-*d*4) spectrum of kaempferol-3-*O*-rhamnoside.

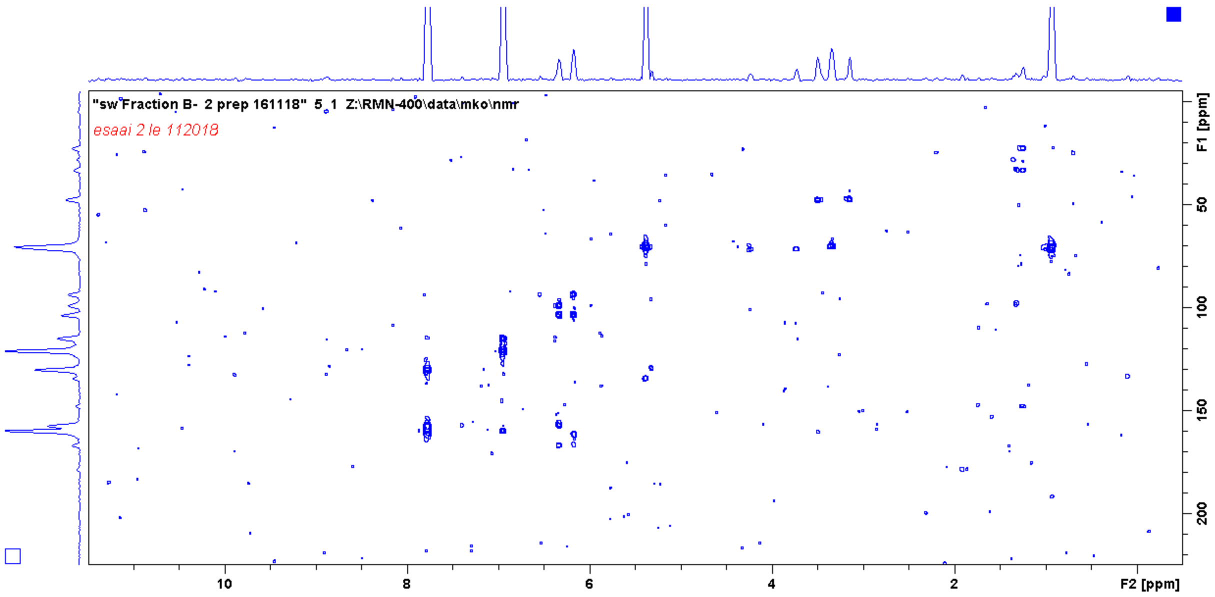

Figure S20. HMBC NMR (400 MHz, MeOD-*d*4) spectrum of kaempferol-3-*O*-rhamnoside.

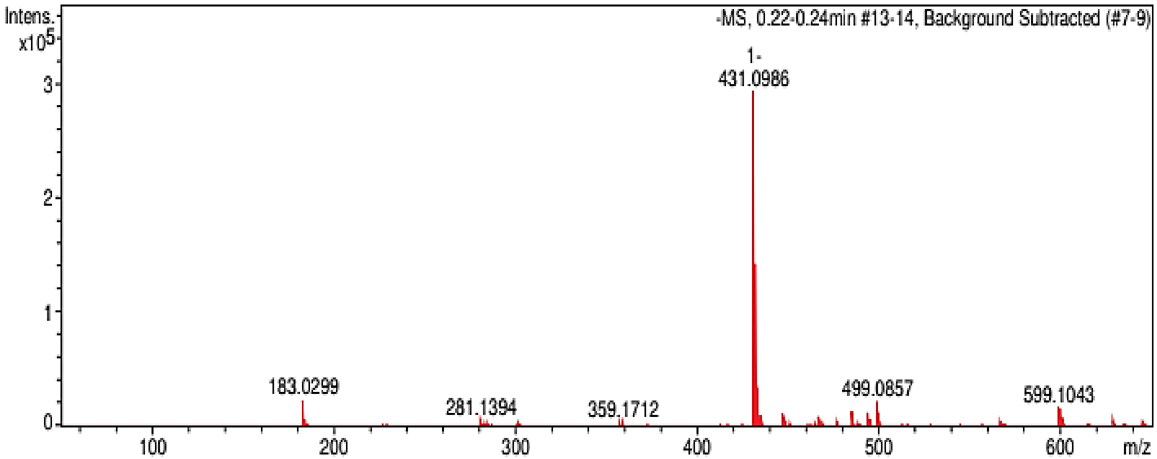

Figure S21. MS (ESI-) spectrum of kaempferol-3-*O*-rhamnoside.

Table S1. NMR data of flavonol glycosides (400 MHz, MeOD-*d*4) (position numbers have been recorded in Figure 4).

**Position**	**The C fraction : Quercetin-3-*O*-rhamnoside**		**The M2 fraction : Myricetin-3-*O*-rhamnoside**		**The B2 fraction : Kaempferol-3-*O*-rhamnoside**	
	**δ ^{1}H**	**COSY**	**δ ^{1}H**	**COSY**	**δ ^{1}H**	**COSY**
6	6.19 s	H8	6.36 d (1.6)	H6	6.19 s	H6
7						
8	6.36 s	H6	6.20 d (1.6)	H8	6.36 s	H8
2′	7.33 d (2.0)	H6′	6.95 s		7.76 d (8.4)	H3′
3′					6.93 d (8.4)	H2′
5′	6.93 d (8.3)	H6′			6.93 d (8.4)	H6′
6′	7.31 dd (8.3, 2.0)	H2′, H5′	6.95 s		7.76 d (8.4)	H5′
1″	5.34 s	H2″	5.31 s	H2″	5.38 d (1.5)	H2″
2″	4.26 m	H1″, H3″	3.80 dd (9.5, 3.2)	H1″, H3″	4.23 dd (3.3, 1.7)	H1″, H3″
3″	3.76 dd (9.4, 3.2)	H2″, H4″	3.55 m	H2″, H4″	3.72 m	H2″, H4″
4″	3.34 dd (9.4, 7.5)	H3″, H5″	3.17 m	H3″, H5″	3.34 m	H3″, H5″
5″	3.41 m	H4″, H6″	3.34 m	H4″, H6″	3.34 m	H4″, H6″
6″	0.94 d (6.1)	H5″	0.84 d (6.1)	H5″	0.93 d (5.7)	H5″

Table S2. 1H-NMR data of loliolide and lupeol (400 MHz, MeOD-*d*4) (position numbers have been recorded in Figure 4).

Position	Lupeol (A12)
	δ ^{1}H
3	3.16 dd (5.4, 10.8)
29	4.68, 4.56, s
30	1.25 s
6 x CH3	1.02, 0.96, 0.94, 0.83, 0.78, 0.76 s

Position	Loliolide (A32)
	δ ^{1}H
2	5.70 s
5	1.98 dt (14.7, 2.4), 1.53 dd (14.7, 3.9)
6	4.33 m
7	2.46 dt (13.7, 2.4), 1.79 dd (13.7, 3.9)
9	1.47, s
10	1.29 s
11	1.79 s
